# Guidance to rational use of pharmaceuticals in gallbladder sarcomatoid carcinoma using patient-derived cancer cells and whole exome sequencing

**DOI:** 10.18632/oncotarget.14146

**Published:** 2016-12-24

**Authors:** Feiling Feng, Qingbao Cheng, Liang Yang, Dadong Zhang, Shunlong Ji, Qiangzu Zhang, Yihui Lin, Fugen Li, Lei Xiong, Chen Liu, Xiaoqing Jiang

**Affiliations:** ^1^ Department of Biliary I, Third Affiliated Hospital of PLA Second Military Medical University, Shanghai, China; ^2^ Division of Translational Medicine, 3D Medicines Corporation, Shanghai, China; ^3^ Changhai Hospital, The Second Military Medical University, Shanghai, China

**Keywords:** gallbladder sarcomatoid carcinoma (GSC), patient-derived cancer cell (PDC), whole exome sequencing (WES), *PIK3CA* amplification, drug sensitivity

## Abstract

**Purpose:**

Gallbladder sarcomatoid carcinoma is a rare cancer with no clinical standard treatment. With the rapid development of next generation sequencing, it has been able to provide reasonable treatment options for patients based on genetic variations. However, most cancer drugs are not approval for gallbladder sarcomatoid carcinoma indications. The correlation between drug response and a genetic variation needs to be further elucidated.

**Experimental Design:**

Three patient-derived cells-JXQ-3D-001, JXQ-3D-002, and JXQ-3D-003, were derived from biopsy samples of one gallbladder sarcomatoid carcinoma patient with progression and have been characterized. In order to study the relationship between drug sensitivity and gene alteration, genetic mutations of three patient-derived cells were discovered by whole exome sequencing, and drug screening has been performed based on the gene alterations and related signaling pathways that are associated with drug targets.

**Results:**

It has been found that there are differences in biological characteristics such as morphology, cell proliferation, cell migration and colony formation activity among these three patient-derived cells although they are derived from the same patient. Their sensitivities to the chemotherapy drugs-Fluorouracil, Doxorubicin, and Cisplatin are distinct. Moreover, none of common chemotherapy drugs could inhibit the proliferations of all three patient-derived cells. Comprehensive analysis of their whole exome sequencing demonstrated that tumor-associated genes *TP53*, *AKT2*, *FGFR3*, *FGF10*, *SDHA*, and *PI3KCA* were mutated or amplified. Part of these alterations are actionable. By screening a set of compounds that are associated with the genetic alteration, it has been found that GDC-0941 and PF-04691502 for PI3K-AKT-mTOR pathway inhibitors could dramatically decrease the proliferation of three patient-derived cells. Importantly, expression of phosphorylated AKT and phosphorylated S6 were markedly decreased after treatments with PI3K-AKT-mTOR pathway inhibitors GDC-0941 (0.5 μM) and PF-04691502 (0.1 μM) in all three patient-derived cells. These data suggested that inhibition of the PI3K-AKT-mTOR pathway that was activated by *PIK3CA* amplification in all three patient-derived cells could reduce the cell proliferation.

**Conclusions:**

A patient-derived cell model combined with whole exome sequencing is a powerful tool to elucidate relationship between drug sensitivities and genetic alternations. In these gallbladder sarcomatoid carcinoma patient-derived cells, it is found that *PIK3CA* amplification could be used as a biomarker to indicate PI3K-AKT-mTOR pathway activation. Block of the pathway may benefit the gallbladder sarcomatoid carcinoma patient with this alternation in hypothesis. The real efficacy needs to be confirmed *in vivo* or in a clinical trial.

## INTRODUCTION

Gallbladder carcinoma (GBC) is the most malignancy in the biliary tract, and ranks fifth among gastrointestinal tract malignancy [1–3]. Gallbladder adenocarcinoma is the dominate type whereas gallbladder sarcomatoid carcinoma (GSC) is rare with epithelial and mesenchymal components [4]. To our best knowledge, only eighty cases of GSC have been reported in literatures worldwide [5–10]. Despite of advances in surgery, radiotherapy, and chemotherapy in past several decades, GSC is still characterized of a poor prognosis with no clinical standard treatment [11, 12]. Up to now, decisions on the elective and therapeutic management of GSC patients are made mainly based on clinical backgrounds, due to lack of targeted drugs approved by the Food and Drug Administration (FDA) or China Food and Drug Administration (CFDA) for GSC [13–15]. Therefore, new targets and methods to guide a rational use of pharmaceuticals are needed for GSC treatment, which could be beneficial for therapeutic management of GSC in clinical application.

The PDC model provides a preclinical tool for translational study with an obvious benefit of infinite supply of a relatively homogeneous cell line that is capable of self-replication [16]. Tumor PDCs established directly from human tumor tissue can serve as an unique way to study the molecular and cellular processes underlying malignant disease and identify novel therapeutic targets [17]. The PDCs have been used to discover drug targets and explore drug resistance in a variety of tumors, such as breast cancer, lung cancer, hepatocellular carcinoma, and etc. [18–22]. However, the PDCs from GSC have not been reported previously.

With the rapid development of the next generation sequencing (NGS), tumor profiling has been widely used in translational medicine research and clinical diagnosis [23–26]. Maolan Li et al provided an insight into the somatic mutational landscape in GBC and highlighted the key role of the ErbB signaling pathway in GBC tumorigenesis using WES [27]. Michele Simbolo et al found that *KRAS* and *TP53* mutations occurred more frequently in GBC than in intrahepatic cholangiocarcinomas (ICC). The molecular subtypes of cholangiocarcinomas were identified and can be explored for targeted drug efficacy in clinical trials [28]. Genotype-based selection of patients for the application of targeted therapies has had a significant impact on the treatment of cancers. Effective targeted therapies, such as tyrosine kinase inhibitors (TKIs) [29], are widely used to treat non–small cell lung cancers (NSCLCs), melanoma, and advanced hepatocellular carcinoma [30–34]. Nevertheless, whether these TKIs can be used for GSC therapy remains unclear. The relationship between the sensitivities of targeted inhibitors and genetic variations in GBC needs to be further elucidated.

In this study, three GSC PDCs that derived from one single GSC patient were established. The biological characteristics and the response to the chemotherapy drugs have been explored. Furthermore, the genetic variations of three GSC PDCs were discovered by WES and their association with the sensitivities of targeted inhibitors has been elucidated.

## RESULTS

### The GSC PDCs preserve some characteristics of the GSC tissue and show heterogeneity inside tumor

The clinical features of a GSC patient were shown in Supplementary Table S1. This GSC patient had an elevated CA19-9 with 95.0 U/mL and total bilirubin of 121.2 μmol/L, which are out of the normal ranges. The Magnetic Resonance Cholangiopanc-reatography (MRCP) and Computed Tomography (CT) on the gallbladder sarcomatoid carcinoma (Supplementary Figure S1) demonstrated interruption of extrahepatic bile duct structure and gallbladder wall thickening, suggesting that the patient might have malignant lesions. The immunohistochemistry staining of the tumor specimens showed pan-cytokeratin (pan-CK) (+), Ki67 (+), Heppar-1 (−), CAM5.2 (+), Vimetin (+), CK19 (−), and SMA (+) (Supplementary Figure S2), which are associated with the clinical characteristics of sarcoma patients [35–38].

**Figure 1:**
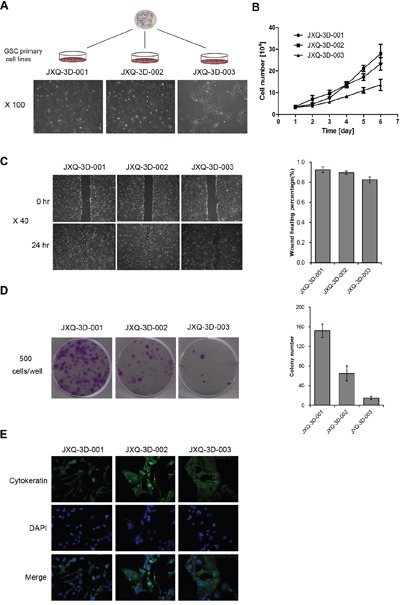
The biological characteristics of three gallbladder sarcomatoid carcinoma patient -derived cell lines (GSC PDCs) The three GSC PDCs (JXQ-3D-001, JXQ-3D-002, and JXQ-3D-003) were derived from the same gallbladder sarcomatoid carcinoma patient. The cell morphology **A**., proliferation **B**., migration **C**., and colony formation activity **D**. of three GSC PDCs were shown. **E**., Cytokeratin (CK) was stained and the images of three GSC PDCs were captured by laser scanning confocal microscopy; Magnification, 100x. Error bars represent mean ± SD from three replicates.

Three GSC PDCs-JXQ-3D-001, JXQ-3D-002 and JXQ-3D-003, were derived from one biopsy of the GSC patient at different sites (Figure 1A). The Short Tandem Repeat (STR) assay identified the karyotype of these PDCs resembling para-carcinoma tissue (Supplementary Table S2). The morphology of three GSC PDCs is different. More biological characteristics of the PDCs including cell proliferation, cell migration, and colony formation activities have been studied (Figure 1B, 1C, 1D). The differences among these PDCs indicated the existence of distinct subclones in this gallbladder sarcomatoid carcinoma tissue. Meanwhile, pan-cytokeratin (CK) expression could be observed in all three GSC PDCs by laser scanning confocal microscopy (Figure 1E). The CK expression in PDCs is consistent with that in the GSC patient tissue by immunohistochemistry staining, indicating that the GSC PDCs preserve some characteristics of this patient's GSC tissue.

### Diverse response of three GSC PDCs to chemotherapy drugs for biliary tract cancer

**Figure 2:**
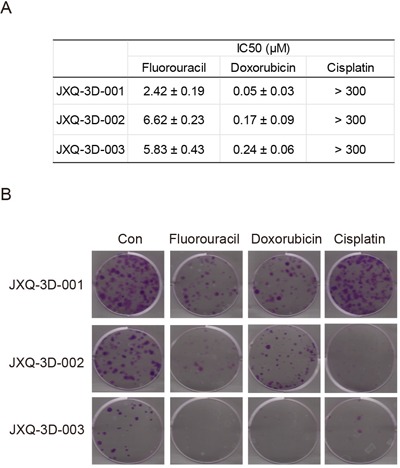
The sensitivities of three GSC PDCs to chemotherapy drugs for tumor of biliary tract **A**. Fifty percent growth inhibitory concentrations (IC50s) of chemotherapy drugs - Fluorouracil, Doxorubicin, and Cisplatin on three GSC PDCs were indicated. This experiment was repeated 3 times and the standard deviation was indicated. **B**. Long-term colony formation assay of GSC PDCs that had been exposed to the indicated drug for 15 days. The concentrations of Fluorouracil, Doxorubicin, and Cisplatin were 5 μM, 0.1 μM, and 150 μM, respectively. Cells were stained using crystal violet.

Three GSC PDCs derived from one single GSC patient can be used as an *in vitro* model to test the effectiveness of chemo-drugs. In this study, Fluorouracil, Doxorubicin and Cisplatin clinically used for GSC treatment were tested in these GSC PDCs for cell proliferation inhibition. All GSC PDCs are not sensitive to Cisplatin with IC50 > 300 μM (Figure 2A). However, Fluorouracil and Doxorubicin are both effective drugs for three GSC PDCs, although IC50 of Doxorubicin is significantly less than that of Fluorouracil (Figure 2A). Colony formation is a better tool to evaluate cell proliferation. To test the effectiveness of chemotherapy drugs on colony formation, Fluorouracil (5 μM), Doxorubicin (0.1 μM) and Cisplatin (150 μM) were applied to each PDC. JXQ-3D-001, JXQ-3D-002 and JXQ-3D-003 cells were treated with three chemotherapy drugs respectively for 10 days and their colonies were evaluated. Fluorouracil and Doxorubicin, not Cisplatin highly inhibit the colony formation of JXQ-3D-001, while Fluorouracil and Cisplatin, not Doxorubicin inhibit the colony formation of JXQ-3D-002. However, all three drugs demonstrated inhibition of JXQ-3D-003 (Figure 2B). These results indicated that a single drug alone could not completely inhibit the proliferation and colony formation of three GSC PDCs from the same patient, which demonstrates the heterogeneity of GSC tumor.

### The variant and CNV landscapes of three GSC PDCs by WES

**Figure 3:**
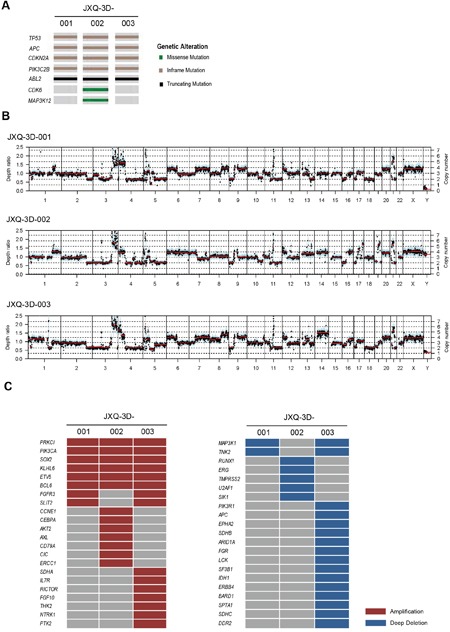
The mutation and CNV landscape of three GSC PDCs detected by whole exome sequencing **A**. The mutational status of tumor-associated genes in three GSC PDCs. **B**. Copy number variation patterns in three GSC PDCs. Y axes denote depth-ratio measurements of coverage obtained in test samples versus a matched blood sample, with assessed copy numbers marked by dashed lines. Each point denotes a genomic region measured by the assay (blue for exons, and cyan for SNPs). All data were ordered by genomic position. Red lines indicate average log-ratio in a segment. **C**. Profile of amplification and deep deletion in three GSC PDCs.

The variant landscapes of three GSC PDCs were further explored to search for appropriate drug targets. To discover the somatic mutations and CNVs for three GSC PDCs, WES of PDCs and their matched blood samples were performed. The mean coverage of three GSC PDCs and blood (BLD) was more than 100 X (Supplementary Table S3). All somatic variants are listed in Supplementary Table S4. Variants from *TP53*, *APC*, *CDKN2A*, *PIK3C2B*, *ABL2*, *CDK6*, and *MAP3K12*, including missense mutation, inframe and truncation, are highly similar across three PDCs (Figure 3A). These PDCs also had similar copy number variation (CNV) patterns (Figure 3B). The gene amplification and deep deletion is listed in Supplementary Table S5. Some of these amplified genes are well-known tumor related genes, such as *AKT2*, *FGFR3*, *FGF10*, *SDHA*, *CCNE1*, *PI3KCA*, and etc (Figure 3C). *AKT2*, *FGFR3*, and *FGF10* amplification had been reported in Chinese gallbladder carcinoma [27]. *PIK3CA* amplification was occasionally observed in breast cancer, gastric cancer and esophageal squamous cell carcinoma [39–43]. *CCNE1*, *SDHA*, and *AKT2* amplifications occurred in only one of three GSC PDCs, while *BCL6* and *PI3KCA* amplifications were detected in all three GSC PDCs. Part of the detected variants and amplifications are actionable.

### Inhibition of the PI3K-AKT-mTOR pathway driven by PIK3CA amplification

**Figure 4:**
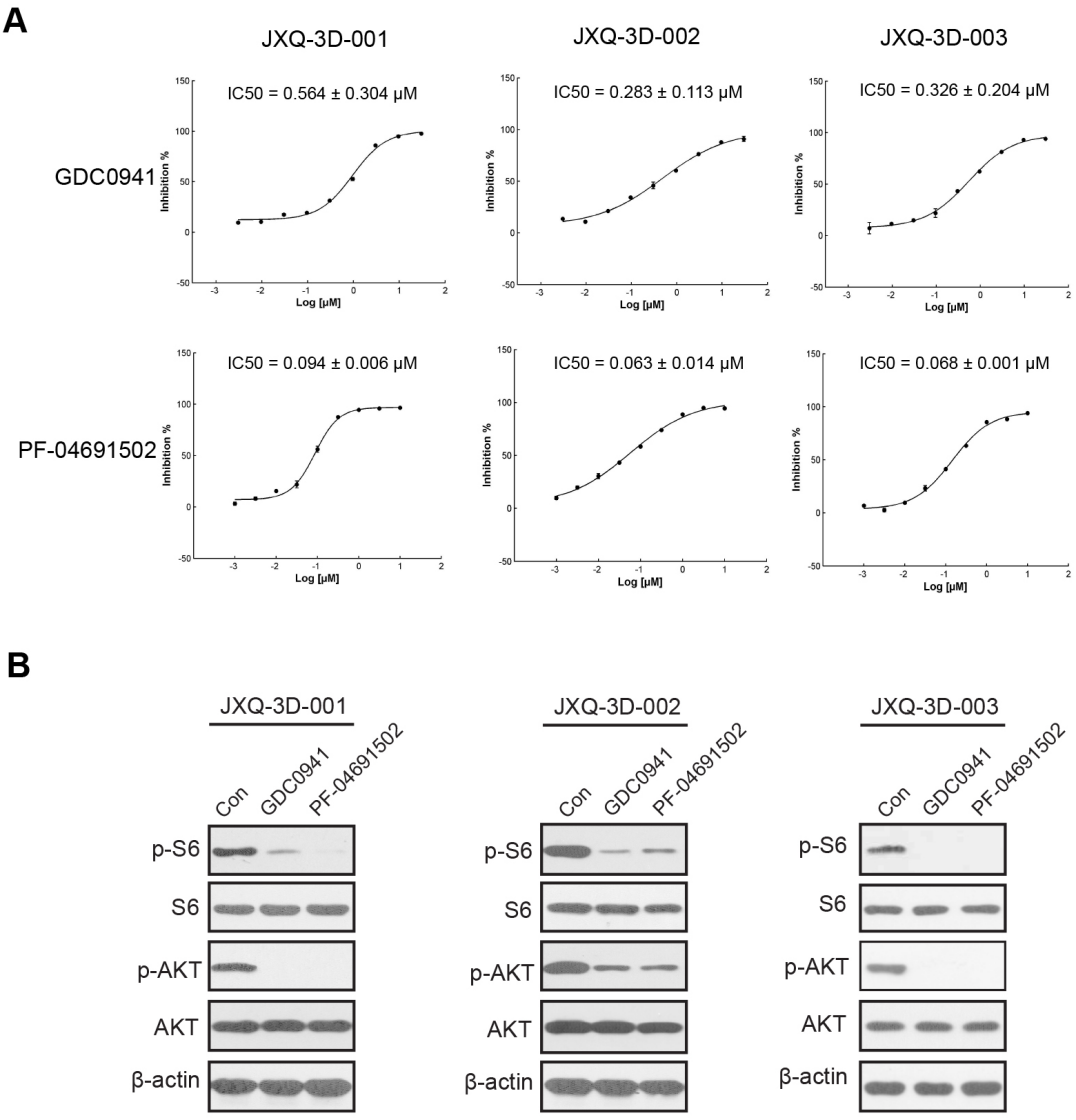
Inhibition of PI3K-AKT-mTOR pathway could reduce the proliferation of three GSC PDCs with PIK3CA amplification **A**. Dose-response curve of PI3K-AKT-mTOR pathway inhibitors in three GSC PDCs (JXQ-3D-001, JXQ-3D-002, and JXQ-3D-003). The IC50s of GDC-0941 and PF-04691502 were shown. **B**. Western blot of PI3K-AKT-mTOR pathway proteins in JXQ-3D-001, JXQ-3D-002, and JXQ-3D-003. Cells were treated with DMSO (Con), GDC-0941 (0.5 μM), or PF-04691502 (0.1 μM) for 6 hours. The expression of indicated proteins were visualized by Western blot using corresponding antibodies.

A set of 9 compounds associated with somatic variants has been screened. IC50s are dramatically different across a set of compounds in these PDCs (Supplementary Table S6). Among these compounds, GDC-0941 and PF-04691502, the PI3K-AKT-mTOR pathway inhibitors, can dramatically inhibit the cell growth of all three GSC PDCs (Figure 4A). Importantly, phosphorylated AKT and phosphorylated S6 were markedly decreased after treatment with GDC-0941 (0.5 μM) and PF-04691502 (0.1 μM) for six hours (Figure 4B). These results suggested that *PIK3CA* amplification in all PDCs may drive the activation of PI3K-AKT-mTOR pathway that can be inhibited by GDC-0941 and PF-04691502.

## DISCUSSION

We have successfully established three GSC PDCs from a single GSC patient. These PDCs showed distinct biological characteristics such as morphology, cell proliferation, cell migration and colony formation. These GSC PDCs also exhibited different sensitivities to the chemotherapy drugs, such as Fluorouracil, Doxorubicin, and Cisplatin. These findings indicated that these PDCs preserve intratumor heterogeneity that has been discovered in other types of tumor [44–48]. In addition, Pan-cytokeratin (CK) expression was detected in all three GSC PDCs, which is concordant with the CK immunohistochemistry staining result in patient GSC tissue. Furthermore, three GSC PDCs remain the molecular features of patient GSC tumor [49] and could be used as *in vitro* model to study the biological features of GSC, which have not been reported in previous studies.

By using WES, genetic mutations, such as *TP53*, *APC*, *CDKN2A* that have been reported previously in GBC [28, 50], were detected in three GSC PDCs. In addition, some mutations that were found in other types of tumor like *CDK6*, *PIK3C2B*, and *ABL2* [51–54] also occurred in our PDCs. Some of the amplified genes in either PDCs are well-known tumor related genes, such as *AKT2*, *FGFR3*, *FGF10*, *SDHA*, and *PI3KCA*. Interestingly, all three GSC PDCs harbor *PIK3CA* amplification that has also been reported in breast cancer, lung adenocarcinoma, and so on [39, 42, 43, 55, 56]. Previous studies indicated that *PIK3CA* amplification might drive PI3K-AKT-mTOR pathway. Our drug screening results in this study supported this hypothesis.

In drug screening, it was found that JXQ-3D-003 with *FGF10* and *FGFR3* amplifications was not sensitive to FGFR pathway inhibitors-AZD4547 and LY2874455 (Figure 3C and Supplementary Table S4). Francesco Iorio, et al also found that the sensitivity of some compounds was not always concordant with genetic variation [57]. Integration of somatic mutations, copy number alterations, DNA methylation, and gene expression together might be a better way to predict drug response. In this study, although *PI3KCA* amplification might be used as a biomarker to elucidate PI3K-AKT-mTOR pathway activation, the response of PDCs to other compounds might not be clearly explained.

Furthermore, our data demonstrated that inhibiting the PI3K-AKT-mTOR pathway could reduce the proliferation of three GSC PDCs. However, the inhibition activity has not been evaluated *in vivo*, because none of three GSC PDCs could develop tumors in nude mice. Although our *in vitro* study suggested that this GSC patient with *PIK3CA* amplification would be benefit from PI3K-AKT-mTOR pathway inhibitors, the true efficacy needs to be confirmed *in vivo* and in a clinical trial.

In conclusion, a tumor PDC model combined with WES has been successfully used to study the relationship between drug sensitivity and genetic variation in GSC. Such a model has provided a foundation for GSC drug target identification and validation. Drug screening in PDCs could elucidate the relationship between a drug and its corresponding target, which would guide a rational use of targeted drugs.

## MATERIALS AND METHODS

### Clinical information of a patient with gallbladder sarcomatoid carcinoma

A 65-year-old male presented with abdominal pain. Laboratory tests showed HCVAb (−), HBsAg (+), HBeAg (−), HBeAb (−), HBcAb (+), CEA (3.1 ng/ml), AFP (4.3 ng/ml) and CA125 (3.1 ng/ml). This patient had an elevated CA19-9 (95.0 U/ml) and his total bilirubin is 121.2μm/L at an abnormal level. Detailed clinical information was described in Supplementary Table S1. Magnetic Resonance Cholangiopancreatography (MRCP) and CT (Supplementary Figure S1) demonstrated interruption of extrahepatic bile duct structure and gallbladder wall thickening, suggesting malignant lesions occurred.

The gallbladder tumor was resected, but a curative resection was impossible due to massive lymphadenopathy of aortic, vena cava and celiac areas. Pathology examination revealed a 4.2 × 3 cm gray mass in the gallbladder neck with (gallbladder) mesenchymal malignant tumor features. Immunohistochemistry (IHC) staining showed the characteristic of this tumor tissue in conformity with the one of leiomyosarcoma (Supplementary Figure S2).

### Patient-derived cells (PDC) derived from a single patient with gallbladder sarcomatoid carcinoma

Fresh gallbladder sarcomatoid carcinoma tissue was placed in culture dishes with proper amount of PBS followed by removal of tissue blood (blood clots), fat, necrotic tissue and connective tissue by ophthalmological forceps and scissors. Subsequently, the cancer tissue was moved into a new dish filled with PBS, and was cut into approximately 1 mm^3^ pieces using ophthalmological forceps and scissors. All pieces were rinsed with PBS for several times and placed into culture flasks with 5 ml of collagenase. After the pieces were digested adequately, the flasks were placed in a shaker at 37°C. When the tissue pieces show good light transmittance and flocculent shape under microscope, they were then centrifuged at 1500 rpm for 5 minutes, and the supernatant was transferred into a 15 ml centrifuge tube. The cell pellets were washed for a few times with PBS and were incubated in DMEM complete culture medium. Lastly, the culture was transferred into a dish and grew until separated cells or cell clusters were visible under an optical microscope. Three GSC PDCs were successfully established from this single patient with gallbladder sarcomatoid carcinoma. Detailed descriptions of three GSC PDCs were shown in Figure 1 and Supplementary Table S2.

### Cell culture and compounds

Three GSC PDCs (JXQ-3D-001, JXQ-3D-002, and JXQ-3D-003) were cultured in DMEM supplemented with 5% FBS. Cells were grown in humidified atmosphere at 5% CO_2_ and 37°C.

Inhibitors for pan-PI3K (GDC-0941), dual PI3K/mTOR (PF-04691502) and chemotherapy drugs (Fluorouracil, Doxorubicin, and Cisplatin) were from Selleckchem group.

### Cell proliferation assay

A cell counter was used to measure cell proliferation. Three GSC PDCs (JXQ-3D-001, JXQ-3D-002, and JXQ-3D-003) were seeded in 6-well plates (one plate per PDC) at the same density. Cells in each well were counted every day for six days in a row.

### Wound-healing assay

Three GSC PDCs (JXQ-3D-001, JXQ-3D-002, and JXQ-3D-003) were grown to confluence, and the wound was introduced by scraping the cell monolayer with a pipette tip p/200. After scraping the wound, the growth medium were changed with the one supplemented with 3% FBS. Each wound was photographed under a light microscope at a magnification of 40X at 0 hour and 24 hours. The assay has been repeated at least three times.

### Colony-Formation Assay

The cells were seeded at 500-800 cells per well in 6-well plates containing complete DMEM on day 0 and incubated at 37°C and 5% CO_2_ for 15 days. On day 15, cells were fixed with 4% polyformaldehyde for 15 min and stained with 1% crystal violet before quantification.

To study the effect of chemotherapy drugs to the colony-formation activities of the three GSC PDCs, cells were seeded as described above. The cells were exposed to Fluorouracil (0 μM, 5 μM), Doxorubicin (0 μM, 0.1 μM), or Cisplatin (0 μM, 150 μM) for 15 days respectively. The cells were fixed and stained as described above. The experiments were triplicated, and the numbers of colonies containing more than 50 cells were microscopically counted.

### Western blot analyses

Three GSC PDCs (JXQ-3D-001, JXQ-3D-002, and JXQ-3D-003) were respectively inoculated in 6-well plates for *in vitro* analyses. When the cells became 70% to 80% confluent, they were treated with 0 and 1μmol/L GDC-0941 or 0 and 0.5μmol/L PF-04691502 for 6 hours. Cells were washed with cold PBS, and lysate was collected using the RIPA protein extraction reagent. The Pierce BCA Protein Assay (Thermo Scientific) was used to quantitate the lysates. Twenty micrograms of protein were loaded and run on 10-well 10% SDS -Tris Gels. All antibodies were incubated in 5% bovine serum albumin (Sigma). Antibodies were obtained from Cell Signaling Technology, or Santa Cruz Biotechnology. The antibodies used were pAKT (Ser473), AKT, pS6 (Ser235/6), p70S6K (S6) and Actin.

### Immunohistochemistry

Clinical samples in FFPE were cut into 5 μm sections and placed on positively-charged slides. Hematoxylin and eosin (HE) stained tissue was analyzed by a pathologist to confirm histology. For IHC of pan-cytokeratin (CK), Ki67, Heppar-1, CAM5.2, Vimetin, CK19 and SMA, slides were deparaffinized and rehydrated. Antigen retrieval was with 10 mM sodium citrate at pH 6.0 under pressure. Slides were washed in PBS. Endogenous peroxidases were blocked with 3% H_2_O_2_ in methanol. For CK, Heppar-1, CAM5.2, CK19 and SMA, slides were blocked with Ctyo-Q immune-diluent (Innovex Biosciences, NB307) followed by primary antibody incubation in Ctyo-Q immune diluent. Antibody concentrations were as follows: pan-Cytokeratin-1:500 (Sant Cruz, sc-81714), HepPar-1-1:100 (Dako, clone OCHIE5), CAM5.2-1:200 (Sigma, 452M-9), Vimetin-1:500 (Abcam, ab137321), CK19-1:500 (Abcam, ab52625) and SMA-1:500 (Gene Tex, GTX100034). After primary antibody, slides were washed in PBS. Primary antibody detection was achieved with Mach 4 HRP polymer (Biocare Medical), followed by 3,3′-diaminobenzidine incubation. Slides were counterstained with Gill's Hematoxylin then washed in water and PBS. Slides were sealed with Universal Mount (Open Biosystems, MBI1232). For Ki-67 (Abgent, AJ1427b), primary antibodies were used at concentrations of 1:200 in 10% normal goat serum. After incubation, slides were washed and blocked with 5% goat serum in 1X PBS. Primary antibody detection was visualized using an anti-rabbit HRP secondary at 1:500 in 5% goat serum (Vector Labs, PI-1000) and DAB substrate. Slides were counterstained as described above.

### Fluorescence microscopy

For studying the expression of pan-cytokeratin (CK) in three GSC PDCs, cells were fixed in 4% paraformaldehyde in PBS for 10 minutes at 37°C. Coverslips were then rinsed three times with PBS, and the cells were permeabilized with PBS containing 0.1% Triton X-100 for 30 minutes at room temperature. After coverslips were rinsed three times with PBS, they were blocked with 5% bovine serum albumin (TBST containing 5% bovine serum albumin) for 15 minutes at room temperature and then incubated with primary antibody (pan-cytokeratin, Sant Cruz, 1:200) for 1 h at room temperature. Then, coverslips were washed three times with TBST and incubated for 30 minutes at room with Alexa-Fluor 488-labelled goat anti-rabbit IgG. After washing three times with TBST, the slides were mounted on VECTASHIELD mounting medium (Vector Laboratories, Burlingame, CA), Images were observed by confocal microscopy using a Leica DMRB (Leica, Solms, Germany).

### The whole exome sequencing

The qualified genomic DNAs were from three GSC PDCs (JXQ-3D-001, JXQ-3D-002, JXQ-3D-003) and this patient blood and were randomly fragmented by Covaris, ligated to Illumina sequenced adapters, and selected for lengths from 150 to 200 bp. Extracted DNAs were then amplified by ligation-mediated PCR (LM-PCR), purified, and hybridized to the NimbleGen SeqCap EZ Exome (44M) array for enrichment. Hybridized fragments were bound to the streptavidin beads, whereas non-hybridized fragments were washed out after 24 hr. We then subjected captured LM-PCR products to the Agilent 2100 Bioanalyzer to estimate the magnitude of enrichment. We independently loaded each captured library from the process described above on three lanes of a NextSeq 500 platform with 100-bp paired-end reads for high-throughput sequencing to ensure that each sample met the desired average coverage. Detailed description of sequencing QC is presented in Supplementary Table S4.

### Analysis of somatic alterations and amplifications/deletions

Sequence reads from three GSC PDCs and BLD genomic DNA were mapped to the reference human genome (hg19) using the Burrows-Wheeler Aligner™ and were processed using Sequence Alignment/Map (SAMtools), Picard pipeline (http://picard.sourceforge.net/) and Genome Analysis Toolkit. Point mutations were identified by MuTect, a Bayesian algorithm; short insertions and deletions determined by local assembly. To detect DNA copy-number alterations (CNAs), we performed SegSeq11 to infer somatic CNA in genomes on the basis of WES reads. Copy numbers < 1.5 were considered to indicate deletions, and those > 2.5 were considered amplifications by comparison to process matched normal controls (BLD). A full list of SNV events and CNAs are presented in Supplementary Table S4 and Supplementary Table S5.

### *In vitro* cell viability studies

For cell viability studies, cells were plated in quadruplicate at a density of 4,000 cells per well in 96-well plates in regular growth medium and allowed to adhere overnight. The compound (Fluorouracil, Doxorubicin, Cisplatin, GDC-0941, PF-04691502 and so on) dose-response was determined by treating with 10 concentrations based on a 3-fold dilution series. Cell viability was measured after 72 hours incubation using the CellTiter-Glo Luminescent Cell Viability Assay (Promega). The concentration of drug resulting in 50% inhibition of cell viability (IC50) was calculated from a four-parameter curve analysis and was determined from a minimum of three experiments. Mean IC50 values and SDs from three experiments with inhibitors are shown in Supplementary Table S6.

### Statistics

Results of compound IC50s are reported as mean values ± standard deviation (SD) from three independent experiments. Colony counts in each group were obtained from the GelCount (Oxford Optronix, United Kingdom) and analyzed using a standard student's T-test.

## SUPPLEMENTARY FIGURES AND TABLES





## Abbreviations

GSC: gallbladder sarcomatoid carcinoma
PDC: patient-derived cell
GBC: gallbladder carcinomas
ICC: intrahepatic cholangiocarcinomas
CNV: copy number variation
MRCP: Magnetic Resonance Cholangiopancreatography
CT: Computed Tomography
CK: cytokeratin
TKI: tyrosine kinase inhibitor
NSCLC: non–small cell lung cancer
IC50: fifty percent growth inhibitory concentration.
