# Supplementary material for: Guidance to rational use of pharmaceuticals in gallbladder sarcomatoid carcinoma using patient-derived cancer cells and whole exome sequencing
Source: Oncotarget. 2016 Dec 24;8(3):5349–60. doi: 10.18632/oncotarget.14146 (PMC5354913; doi:10.18632/oncotarget.14146)
Supplement: Supplementary file 1 [file oncotarget-08-5349-s001.pdf]

Guidance to rational use of pharmaceuticals in gallbladder sarcomatoid carcinoma using patient-derived cancer cells and whole exome sequencing

SUPPLEMENTARY FIGURES AND TABLES

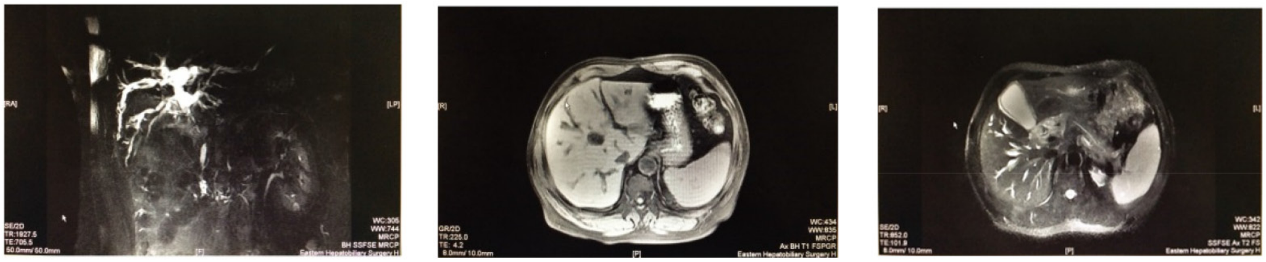

Supplementary Figure S1: The results of Magnetic Resonance Cholangiopancreatography (MRCP) and Computed Tomography (CT) on this gallbladder sarcomatoid carcinoma patient. Interruption of extrahepatic bile duct structure and gallbladder wall thickening was observed

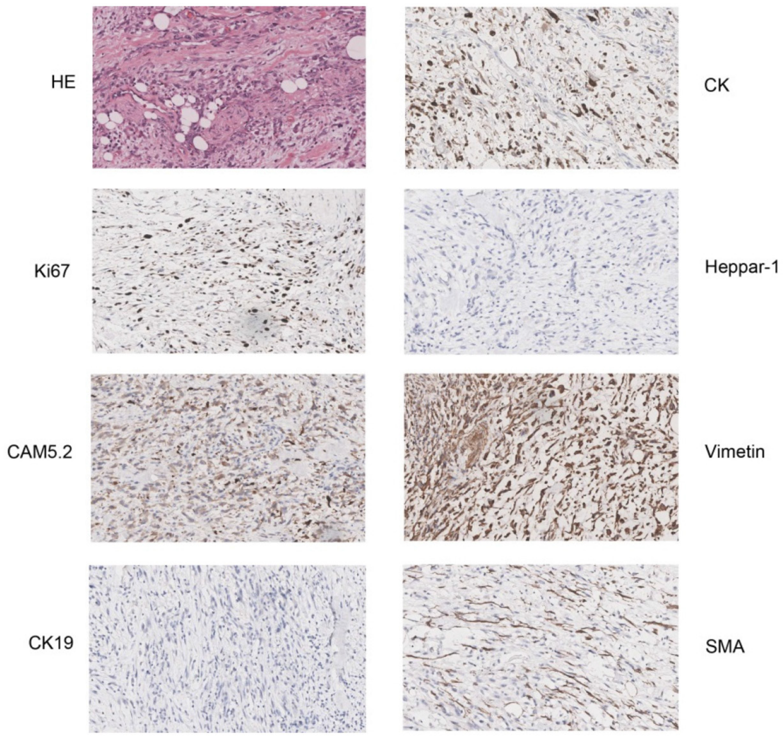

Supplementary Figure S2: Hematein Eosin (HE) staining and immunohistochemical profiles of clinical markers in this gallbladder sarcomatoid carcinoma sample. Representative examples of immunohistochemical staining for pan-Cytokeratin (CK), Ki67, Heppar-1, CAM5, Vimetin, CK19, and SMA on gallbladder sarcomatoid carcinoma. The prevalence of positive cases within the different tumor types were shown. Original magnfications 20 x.

Supplementary Table S1: Patient characteristics

Patient ID	Gender	Age	HCVAb	HBsAg	HBeAg	HBeAb	HbcAb	CEA (ng/ml)	CA19-9 (U/ml)	CA125 (U/ml)	CA72-4 (U/ml)	AFP (ng/ml)
138816	Male	65	Negative	Positive	Negative	Negative	Positive	3.1	95.0	17.9	1.0	4.3

Total bilirubin (μmol/L)	TBA (μmol/L)	Tumor encapsulation	Microscopic vascular invasion
121.2	19.9	No	No

Supplementary Table S2: The Short Tandem Repeat (STR) of the three GSC PDCs and para-carcinoma tissue

	Para-carcinoma tissue		JXQ-3D-001		JXQ-3D-002		JXQ-3D-003	
**D5S818**	11	11	11	11	11	11	11	11
**D13S317**	11	12	11	12	11	12	11	12
**D7S820**	10	12	10	12	10	12	10	12
**D16S539**	10	11	10	11	10	11	10	11
**VWA**	16	19	19	19	19	19	19	19
**THCC01**	7	7	7	7	7	7	7	7
**AMEL**	X	Y	X	Y	X	X	X	Y
**TPOX**	8	11	8	11	8	11	8	11
**CSF1PO**	9	11	9	9	9	9	9	9
**D12S391**	17	18	17	17	17	17	17	17
**FGA**	19	20	19	19	19	19	19	19
**D2S1338**	16	19	16	19	16	19	16	19
**D21S11**	29	32.2	29	32.2	29	32.2	29	32.2
**D18S51**	14	16	14	16	14	16	14	16
**D8S1179**	10	15	10	15	10	15	10	15
**D3S1358**	15	15	15	15	15	15	15	15
**D6S1043**	12	14	12	14	12	14	12	14
**PENTAE**	5	11	5	11	5	11	5	11
**D19S433**	13.2	14	13.2	13.2	13.2	13.2	13.2	13.2
**PENTAD**	9	13	9	9	9	9	9	9

Supplementary Table S3: The mean coverage of whole exome sequencing in three GSC PDCs samples

Sample	Mean coverage	Duplicate rate	On target (%)	On flanking (%)	Off target (%)
**BLD**	100.22	0.12	52.49	27.71	19.80
**JXQ-3D-001**	107.32	0.13	53.79	26.91	19.30
**JXQ-3D-002**	101.49	0.13	54.39	26.14	19.48
**JXQ-3D-003**	105.92	0.13	53.99	26.65	19.37

Supplementary Table S4: The SNV events of the three GSC PDCs

See Supplementary File 1

Supplementary Table S5: The genes with amplifications and deep deletions in three GSC PDCs

	JXQ-3D-001	JXQ-3D-002	JXQ-3D-003
**Genes with amplifications**	PRKCI	PRKCI	PRKCI
	PIK3CA	PIK3CA	PIK3CA
	SOX2	SOX2	SOX2
	KLHL6	KLHL6	KLHL6
	ETV5	ETV5	ETV5
	BCL6	BCL6	BCL6
	FGFR3	CCNE1	FGFR3
	SLIT2	CEBPA	SLIT2
		AKT2	SDHA
		AXL	IL7R
		CD79A	RICTOR
		CIC	FGF10
		ERCC1	THK2
			NTRK1
			PTK2
**Genes with deep deletions**	MAP3K1	RUNX1	MAP3K1
	TNK2	ERG	TNK2
		TMPRSS2	PIK3R1
		U2AF1	APC
		SIK1	EPHA2
			SDHB
			ARID1A
			FGR
			LCK
			SF3B1
			IDH1
			ERBB4
			BARD1
			SPTA1
			SDHC
			DDR2

Supplementary Table S6: The mean IC50 values and SDs from three experiments with inhibitors

Drug name	Target	IC50(μM)		
		JXQ-3D-001	JXQ-3D-002	JXQ-3D-003
GDC0941	pan-PI3K	0.564 ± 0.304	0.283 ± 0.113	0.326 ± 0.204
PF-04691502	PI3K/mTOR	0.094 ± 0.006	0.063 ± 0.014	0.068 ± 0.001
AZD4547	FGFR1/2/3	0.330 ± 0.108	> 10	> 10
LY2874455	FGFR1/2/3/4	0.142 ± 0.079	1.333 ± 0.266	1.792 ± 0.589
LEE011	CDK4/6	>30	>30	>30
AXL-1717	IGF1R	0.217 ± 0.008	0.286 ± 0.008	0.290 ± 0.003
ABT-888	PARP1, PARP2	11.102 ± 1.080	8.920 ± 2.973	>30
Sorafenib	intracellular CRAF, BRAF, mutant BRAF	6.978 ± 0.092	7.689 ± 0.073	6.835 ± 0.008
Vemurafenib	BRAF	2.206 ± 0.053	5.503 ± 0.384	5.630 ± 0.694
