# Supplementary material for: Guidance to rational use of pharmaceuticals in gallbladder sarcomatoid carcinoma using patient-derived cancer cells and whole exome sequencing
Source: Oncotarget. 2016 Dec 24;8(3):5349–60. doi: 10.18632/oncotarget.14146 (PMC5354913; doi:10.18632/oncotarget.14146)
Supplement: Supplementary file 2 [file oncotarget-08-5349-s002.docx]

**Table S4. The SNV events of the three GSC PDCs**

| **Chr** | **Start** | **End** | **Ref** | **Alt** | **Func.refGene** | **Gene.refGene** | **ExonicFunc.refGene** | **JXQ-3D-001** | **JXQ-3D-002** | **JXQ-3D-003** |
| --- | --- | --- | --- | --- | --- | --- | --- | --- | --- | --- |
| 5 | 112176098 | 112176098 | C | - | exonic | APC | frameshift deletion | 1 | 1 | 1 |
| 15 | 30010759 | 30010759 | G | A | exonic | TJP1 | nonsynonymous SNV | 1 | 1 | 1 |
| 6 | 152784544 | 152784544 | G | T | exonic | SYNE1 | nonsynonymous SNV | 1 | 1 | 1 |
| 12 | 21968750 | 21968750 | T | C | exonic | ABCC9 | nonsynonymous SNV | 1 | 1 | 1 |
| 7 | 45124347 | 45124347 | C | T | exonic | NACAD | nonsynonymous SNV | 1 | 1 | 1 |
| 8 | 91929834 | 91929834 | G | A | exonic | NECAB1 | nonsynonymous SNV | 1 | 1 | 1 |
| 17 | 7578246 | 7578246 | - | A | exonic | TP53 | frameshift insertion | 1 | 1 | 1 |
| 20 | 25388499 | 25388499 | C | T | exonic | GINS1 | nonsynonymous SNV | 1 | 1 | 1 |
| 14 | 103986867 | 103986867 | G | A | exonic | CKB | nonsynonymous SNV | 1 | 1 | 1 |
| 1 | 204438529 | 204438529 | T | - | exonic | PIK3C2B | frameshift deletion | 1 | 1 | 1 |
| 9 | 78601109 | 78601109 | G | A | exonic | PCSK5 | nonsynonymous SNV | 1 | 1 | 1 |
| 6 | 79752705 | 79752705 | G | C | exonic | PHIP | stopgain SNV | 1 | 1 | 1 |
| 1 | 149858166 | 149858166 | G | A | exonic | HIST2H2BE | nonsynonymous SNV | 1 | 1 | 1 |
| 12 | 101745860 | 101745860 | G | C | exonic | UTP20 | nonsynonymous SNV | 1 | 1 | 1 |
| 14 | 102505808 | 102505808 | - | T | exonic | DYNC1H1 | frameshift insertion | 1 | 1 | 1 |
| 12 | 116444125 | 116444125 | G | C | exonic | MED13L | nonsynonymous SNV | 1 | 1 | 1 |
| 12 | 40645267 | 40645267 | G | A | exonic | LRRK2 | nonsynonymous SNV | 1 | 1 | 1 |
| 6 | 76407234 | 76407234 | G | A | splicing | SENP6 | NAN | 1 | 1 | 1 |
| 9 | 842042 | 842042 | G | C | exonic | DMRT1 | nonsynonymous SNV | 1 | 1 | 1 |
| 16 | 58592436 | 58592436 | G | A | exonic | CNOT1 | nonsynonymous SNV | 1 | 1 | 1 |
| 12 | 123022969 | 123022969 | C | T | exonic | KNTC1 | nonsynonymous SNV | 1 | 1 | 1 |
| 12 | 6601542 | 6601542 | C | G | exonic | MRPL51 | nonsynonymous SNV | 1 | 1 | 1 |
| 17 | 40831743 | 40831743 | T | C | exonic | CCR10 | nonsynonymous SNV | 1 | 1 | 1 |
| 20 | 30538178 | 30538178 | C | T | exonic | PDRG1 | nonsynonymous SNV | 1 | 1 | 1 |
| 1 | 117142603 | 117142603 | C | G | exonic | IGSF3 | nonsynonymous SNV | 1 | 1 | 1 |
| 8 | 100133680 | 100133680 | C | G | exonic | VPS13B | nonsynonymous SNV | 1 | 1 | 1 |
| 19 | 52869988 | 52869988 | G | A | exonic | ZNF610 | nonsynonymous SNV | 1 | 1 | 1 |
| 10 | 69682756 | 69682756 | C | G | exonic | HERC4 | nonsynonymous SNV | 1 | 1 | 1 |
| 8 | 100133670 | 100133670 | C | G | exonic | VPS13B | nonsynonymous SNV | 1 | 1 | 1 |
| 2 | 145161674 | 145161674 | C | T | exonic | ZEB2 | nonsynonymous SNV | 1 | 1 | 1 |
| 18 | 10532737 | 10532737 | G | C | exonic | NAPG | nonsynonymous SNV | 1 | 1 | 1 |
| 13 | 99992802 | 99992802 | C | G | exonic | UBAC2 | nonsynonymous SNV | 1 | 1 | 1 |
| 22 | 26771604 | 26771604 | C | T | exonic | SEZ6L | nonsynonymous SNV | 1 | 1 | 1 |
| 1 | 153935131 | 153935131 | G | T | exonic | SLC39A1 | nonsynonymous SNV | 1 | 1 | 1 |
| 7 | 97841865 | 97841865 | G | A | exonic | BHLHA15 | nonsynonymous SNV | 1 | 1 | 1 |
| 15 | 22945103 | 22945103 | G | A | exonic | CYFIP1 | nonsynonymous SNV | 1 | 1 | 1 |
| 3 | 169854311 | 169854311 | C | T | exonic | PHC3 | nonsynonymous SNV | 1 | 1 | 1 |
| 8 | 120793313 | 120793313 | G | A | exonic | TAF2 | stopgain SNV | 1 | 1 | 1 |
| 16 | 81209316 | 81209316 | G | A | exonic | PKD1L2 | nonsynonymous SNV | 1 | 1 | 1 |
| 4 | 4281428 | 4281428 | C | T | exonic | LYAR | nonsynonymous SNV | 1 | 1 | 1 |
| 8 | 57026442 | 57026442 | G | T | exonic | MOS | nonsynonymous SNV | 1 | 1 | 1 |
| X | 26212858 | 26212858 | G | T | exonic | MAGEB6 | nonsynonymous SNV | 1 | 1 | 1 |
| 19 | 3910832 | 3910832 | G | A | exonic | ATCAY | nonsynonymous SNV | 1 | 1 | 1 |
| 19 | 10610523 | 10610523 | C | T | exonic | KEAP1 | nonsynonymous SNV | 1 | 1 | 1 |
| 5 | 149210373 | 149210373 | C | T | exonic | PPARGC1B | nonsynonymous SNV | 1 | 1 | 1 |
| 3 | 108627044 | 108627044 | A | G | exonic | GUCA1C | nonsynonymous SNV | 1 | 1 | 1 |
| 19 | 40023322 | 40023322 | G | A | exonic | EID2B | nonsynonymous SNV | 1 | 1 | 1 |
| 17 | 40832446 | 40832446 | C | T | exonic | CCR10 | nonsynonymous SNV | 1 | 1 | 1 |
| 7 | 148963810 | 148963810 | G | A | exonic | ZNF783 | nonsynonymous SNV | 1 | 1 | 1 |
| 17 | 79663885 | 79663885 | T | G | exonic | HGS | nonsynonymous SNV | 1 | 1 | 1 |
| 11 | 65408664 | 65408664 | A | C | exonic | SIPA1 | nonsynonymous SNV | 1 | 1 | 1 |
| 1 | 144994659 | 144994659 | G | A | exonic | PDE4DIP | nonsynonymous SNV | 1 | 1 | 1 |
| 1 | 179084097 | 179084097 | G | A | exonic | ABL2 | stopgain SNV | 1 | 1 | 0 |
| 2 | 219082246 | 219082246 | A | T | exonic | ARPC2 | nonsynonymous SNV | 1 | 1 | 0 |
| 9 | 125330191 | 125330191 | G | C | exonic | OR1L8 | nonsynonymous SNV | 1 | 0 | 1 |
| 2 | 84924698 | 84924698 | G | A | splicing | DNAH6 | NAN | 1 | 0 | 1 |
| 9 | 125330065 | 125330065 | G | C | exonic | OR1L8 | nonsynonymous SNV | 1 | 0 | 1 |
| 4 | 70918816 | 70918816 | T | C | exonic | HTN1 | nonsynonymous SNV | 1 | 0 | 1 |
| 12 | 53585798 | 53585798 | C | T | exonic | ITGB7 | nonsynonymous SNV | 1 | 0 | 1 |
| 16 | 24372750 | 24372750 | A | T | exonic | CACNG3 | nonsynonymous SNV | 1 | 0 | 1 |
| 9 | 138642853 | 138642853 | G | A | exonic | KCNT1 | nonsynonymous SNV | 1 | 0 | 1 |
| 2 | 125281920 | 125281920 | C | G | exonic | CNTNAP5 | nonsynonymous SNV | 1 | 0 | 1 |
| 11 | 6913608 | 6913608 | T | G | exonic | OR2D2 | nonsynonymous SNV | 1 | 0 | 1 |
| 9 | 116811482 | 116811482 | C | T | exonic | ZNF618 | nonsynonymous SNV | 1 | 0 | 1 |
| 12 | 53586141 | 53586141 | C | G | exonic | ITGB7 | nonsynonymous SNV | 1 | 0 | 1 |
| 9 | 100204061 | 100204061 | G | A | exonic | TDRD7 | stopgain SNV | 1 | 0 | 1 |
| 19 | 9449901 | 9449901 | C | G | exonic | ZNF559 | nonsynonymous SNV | 1 | 0 | 1 |
| 4 | 20255512 | 20255512 | C | T | exonic | SLIT2 | nonsynonymous SNV | 1 | 0 | 1 |
| 3 | 186510646 | 186510646 | C | T | exonic | RFC4 | nonsynonymous SNV | 1 | 0 | 1 |
| 17 | 73498786 | 73498786 | T | G | exonic | CASKIN2 | nonsynonymous SNV | 1 | 0 | 1 |
| 1 | 12266998 | 12266998 | C | T | exonic | TNFRSF1B | nonsynonymous SNV | 1 | 0 | 0 |
| 1 | 152883174 | 152883174 | A | G | exonic | IVL | nonsynonymous SNV | 1 | 0 | 0 |
| 9 | 139090512 | 139090512 | T | A | exonic | LHX3 | nonsynonymous SNV | 1 | 0 | 0 |
| 20 | 62851156 | 62851156 | A | C | exonic | MYT1 | nonsynonymous SNV | 1 | 0 | 0 |
| 8 | 124268047 | 124268047 | G | C | exonic | ZHX1 | stopgain SNV | 1 | 0 | 0 |
| 9 | 133961009 | 133961009 | A | C | exonic | LAMC3 | nonsynonymous SNV | 1 | 0 | 0 |
| 17 | 70119801 | 70119801 | T | G | exonic | SOX9 | nonsynonymous SNV | 1 | 0 | 0 |
| 4 | 3449697 | 3449697 | A | C | exonic | HGFAC | nonsynonymous SNV | 1 | 0 | 0 |
| 20 | 62624765 | 62624765 | A | T | exonic | PRPF6 | nonsynonymous SNV | 1 | 0 | 0 |
| 3 | 172166121 | 172166121 | T | G | exonic | GHSR | nonsynonymous SNV | 1 | 0 | 0 |
| 1 | 85575761 | 85575761 | G | T | exonic | WDR63 | nonsynonymous SNV | 1 | 0 | 0 |
| 12 | 111020769 | 111020769 | T | G | exonic | PPTC7 | nonsynonymous SNV | 1 | 0 | 0 |
| 19 | 36322199 | 36322199 | G | A | exonic | NPHS1 | nonsynonymous SNV | 1 | 0 | 0 |
| 6 | 15497224 | 15497224 | A | C | exonic | JARID2 | nonsynonymous SNV | 1 | 0 | 0 |
| 8 | 59515843 | 59515843 | T | G | exonic | NSMAF | nonsynonymous SNV | 1 | 0 | 0 |
| 6 | 32411628 | 32411628 | A | T | exonic | HLA-DRA | nonsynonymous SNV | 1 | 0 | 0 |
| 20 | 35433142 | 35433142 | G | A | exonic | SOGA1 | nonsynonymous SNV | 1 | 0 | 0 |
| 15 | 85164309 | 85164309 | A | T | exonic | ZSCAN2 | nonsynonymous SNV | 1 | 0 | 0 |
| X | 153695756 | 153695756 | A | C | exonic | PLXNA3 | nonsynonymous SNV | 1 | 0 | 0 |
| 20 | 61867704 | 61867704 | T | G | exonic | BIRC7 | nonsynonymous SNV | 1 | 0 | 0 |
| 7 | 48450201 | 48450201 | T | G | exonic | ABCA13 | nonsynonymous SNV | 1 | 0 | 0 |
| 9 | 139264987 | 139264987 | T | G | exonic | CARD9 | nonsynonymous SNV | 1 | 0 | 0 |
| 5 | 145719744 | 145719744 | T | A | exonic | POU4F3 | nonsynonymous SNV | 1 | 0 | 0 |
| 1 | 6520091 | 6520091 | T | A | exonic | ESPN | nonsynonymous SNV | 1 | 0 | 0 |
| 7 | 123672576 | 123672576 | A | C | exonic | TMEM229A | nonsynonymous SNV | 1 | 0 | 0 |
| 19 | 8563494 | 8563494 | A | C | exonic | PRAM1 | nonsynonymous SNV | 1 | 0 | 0 |
| 1 | 9794071 | 9794071 | C | T | exonic | CLSTN1 | nonsynonymous SNV | 1 | 0 | 0 |
| 12 | 657252 | 657252 | A | C | exonic | B4GALNT3 | nonsynonymous SNV | 1 | 0 | 0 |
| 19 | 8619566 | 8619566 | T | G | exonic | MYO1F | nonsynonymous SNV | 1 | 0 | 0 |
| 1 | 150531481 | 150531481 | T | G | exonic | ADAMTSL4 | nonsynonymous SNV | 1 | 0 | 0 |
| 18 | 63476953 | 63476953 | T | A | exonic | CDH7 | nonsynonymous SNV | 1 | 0 | 0 |
| 4 | 947038 | 947038 | T | G | exonic | TMEM175 | nonsynonymous SNV | 1 | 0 | 0 |
| 1 | 156639488 | 156639488 | A | C | exonic | NES | nonsynonymous SNV | 1 | 0 | 0 |
| 11 | 5080841 | 5080841 | T | G | exonic | OR52E2 | nonsynonymous SNV | 1 | 0 | 0 |
| 11 | 11354293 | 11354293 | A | C | exonic | GALNT18 | nonsynonymous SNV | 1 | 0 | 0 |
| 6 | 42227136 | 42227136 | T | G | exonic | TRERF1 | nonsynonymous SNV | 1 | 0 | 0 |
| 1 | 156146420 | 156146420 | G | T | exonic | SEMA4A | nonsynonymous SNV | 1 | 0 | 0 |
| 16 | 1664810 | 1664810 | A | C | exonic | CRAMP1L | nonsynonymous SNV | 1 | 0 | 0 |
| 1 | 156348061 | 156348061 | T | G | exonic | RHBG | nonsynonymous SNV | 1 | 0 | 0 |
| 12 | 101728228 | 101728228 | A | T | exonic | UTP20 | nonsynonymous SNV | 1 | 0 | 0 |
| 19 | 5214398 | 5214398 | A | T | exonic | PTPRS | nonsynonymous SNV | 1 | 0 | 0 |
| 15 | 68619047 | 68619047 | G | A | exonic | ITGA11 | nonsynonymous SNV | 1 | 0 | 0 |
| 17 | 80863868 | 80863868 | T | G | exonic | TBCD | nonsynonymous SNV | 1 | 0 | 0 |
| 6 | 42227119 | 42227119 | T | G | exonic | TRERF1 | nonsynonymous SNV | 1 | 0 | 0 |
| 10 | 7605326 | 7605326 | G | A | exonic | ITIH5 | nonsynonymous SNV | 1 | 0 | 0 |
| 19 | 41798355 | 41798355 | A | T | exonic | HNRNPUL1 | nonsynonymous SNV | 1 | 0 | 0 |
| X | 129149305 | 129149305 | A | C | exonic | BCORL1 | nonsynonymous SNV | 1 | 0 | 0 |
| 5 | 146775194 | 146775194 | A | C | exonic | DPYSL3 | nonsynonymous SNV | 1 | 0 | 0 |
| 15 | 94943223 | 94943223 | A | G | exonic | MCTP2 | nonsynonymous SNV | 1 | 0 | 0 |
| 17 | 73725402 | 73725402 | G | T | exonic | ITGB4 | nonsynonymous SNV | 1 | 0 | 0 |
| 11 | 63283774 | 63283774 | T | G | exonic | LGALS12 | nonsynonymous SNV | 1 | 0 | 0 |
| 12 | 21644462 | 21644462 | T | A | exonic | RECQL | nonsynonymous SNV | 1 | 0 | 0 |
| 2 | 85097596 | 85097596 | T | G | exonic | TRABD2A | nonsynonymous SNV | 1 | 0 | 0 |
| 2 | 97474298 | 97474298 | A | C | exonic | CNNM4 | nonsynonymous SNV | 1 | 0 | 0 |
| 6 | 42073041 | 42073041 | T | G | exonic | C6orf132 | nonsynonymous SNV | 1 | 0 | 0 |
| X | 54955807 | 54955807 | A | T | exonic | TRO | nonsynonymous SNV | 1 | 0 | 0 |
| 22 | 24573681 | 24573681 | T | A | exonic | CABIN1 | nonsynonymous SNV | 1 | 0 | 0 |
| 8 | 22438923 | 22438923 | A | C | exonic | PDLIM2 | nonsynonymous SNV | 1 | 0 | 0 |
| 8 | 98788035 | 98788035 | T | G | exonic | LAPTM4B | nonsynonymous SNV | 1 | 0 | 0 |
| 19 | 6307284 | 6307284 | A | T | exonic | ACER1 | nonsynonymous SNV | 1 | 0 | 0 |
| 12 | 6923400 | 6923400 | G | C | exonic | CD4 | nonsynonymous SNV | 1 | 0 | 0 |
| 9 | 139379297 | 139379297 | T | A | exonic | C9orf163 | nonsynonymous SNV | 1 | 0 | 0 |
| X | 54837368 | 54837368 | T | G | exonic | MAGED2 | nonsynonymous SNV | 1 | 0 | 0 |
| 22 | 18566390 | 18566390 | T | G | exonic | PEX26 | nonsynonymous SNV | 1 | 0 | 0 |
| 9 | 139902960 | 139902960 | A | C | exonic | ABCA2 | nonsynonymous SNV | 1 | 0 | 0 |
| 17 | 45925350 | 45925350 | T | A | exonic | SP6 | nonsynonymous SNV | 1 | 0 | 0 |
| X | 38664333 | 38664333 | A | C | exonic | MID1IP1 | nonsynonymous SNV | 1 | 0 | 0 |
| 1 | 150956483 | 150956483 | A | C | exonic | ANXA9 | nonsynonymous SNV | 1 | 0 | 0 |
| 1 | 20517885 | 20517885 | A | T | exonic | UBXN10 | nonsynonymous SNV | 1 | 0 | 0 |
| 4 | 113435977 | 113435977 | T | G | exonic | NEUROG2 | nonsynonymous SNV | 1 | 0 | 0 |
| 17 | 76201740 | 76201740 | A | C | exonic | AFMID | nonsynonymous SNV | 1 | 0 | 0 |
| 6 | 33647747 | 33647747 | A | C | exonic | ITPR3 | nonsynonymous SNV | 1 | 0 | 0 |
| X | 153217555 | 153217555 | T | G | exonic | HCFC1 | nonsynonymous SNV | 1 | 0 | 0 |
| 17 | 9590188 | 9590188 | A | T | exonic | USP43 | stopgain SNV | 1 | 0 | 0 |
| X | 152991502 | 152991502 | T | G | exonic | ABCD1 | nonsynonymous SNV | 1 | 0 | 0 |
| 8 | 10465465 | 10465465 | G | T | exonic | RP1L1 | nonsynonymous SNV | 1 | 0 | 0 |
| 3 | 97367170 | 97367170 | C | T | exonic | EPHA6 | nonsynonymous SNV | 1 | 0 | 0 |
| 19 | 2217057 | 2217057 | T | G | exonic | DOT1L | nonsynonymous SNV | 1 | 0 | 0 |
| 2 | 112939423 | 112939423 | A | C | exonic | FBLN7 | nonsynonymous SNV | 1 | 0 | 0 |
| 14 | 22038838 | 22038838 | A | T | exonic | OR10G3 | nonsynonymous SNV | 1 | 0 | 0 |
| 3 | 52324428 | 52324428 | T | G | exonic | GLYCTK | nonsynonymous SNV | 1 | 0 | 0 |
| 18 | 11148574 | 11148574 | A | T | exonic | PIEZO2 | nonsynonymous SNV | 1 | 0 | 0 |
| 16 | 70698588 | 70698588 | T | G | exonic | MTSS1L | nonsynonymous SNV | 1 | 0 | 0 |
| 18 | 44260240 | 44260240 | A | C | exonic | ST8SIA5 | nonsynonymous SNV | 1 | 0 | 0 |
| 19 | 56206176 | 56206176 | A | C | exonic | EPN1 | nonsynonymous SNV | 1 | 0 | 0 |
| 2 | 74757227 | 74757227 | A | C | exonic | HTRA2 | nonsynonymous SNV | 1 | 0 | 0 |
| 6 | 39267507 | 39267507 | T | G | exonic | KCNK17 | nonsynonymous SNV | 1 | 0 | 0 |
| 9 | 104499682 | 104499682 | A | C | exonic | GRIN3A | nonsynonymous SNV | 1 | 0 | 0 |
| 19 | 36220975 | 36220975 | G | C | exonic | KMT2B | nonsynonymous SNV | 1 | 0 | 0 |
| 7 | 102312019 | 102312019 | G | T | exonic | POLR2J2 | nonsynonymous SNV | 1 | 0 | 0 |
| 17 | 73235963 | 73235963 | T | G | exonic | GGA3 | nonsynonymous SNV | 1 | 0 | 0 |
| 19 | 40433740 | 40433740 | A | C | exonic | FCGBP | nonsynonymous SNV | 1 | 0 | 0 |
| 1 | 16464615 | 16464615 | T | G | exonic | EPHA2 | nonsynonymous SNV | 1 | 0 | 0 |
| 17 | 73872873 | 73872873 | T | G | exonic | TRIM47 | nonsynonymous SNV | 1 | 0 | 0 |
| 22 | 22317259 | 22317259 | T | G | exonic | TOP3B | nonsynonymous SNV | 1 | 0 | 0 |
| 5 | 133474659 | 133474659 | A | C | exonic | TCF7 | nonsynonymous SNV | 1 | 0 | 0 |
| 1 | 21553685 | 21553685 | C | A | exonic | ECE1 | nonsynonymous SNV | 1 | 0 | 0 |
| 1 | 110051511 | 110051511 | T | G | exonic | AMIGO1 | nonsynonymous SNV | 1 | 0 | 0 |
| 2 | 197541335 | 197541335 | G | T | exonic | CCDC150 | nonsynonymous SNV | 1 | 0 | 0 |
| 5 | 172586998 | 172586998 | T | G | exonic | BNIP1 | nonsynonymous SNV | 1 | 0 | 0 |
| 14 | 36004639 | 36004639 | A | C | exonic | INSM2 | nonsynonymous SNV | 1 | 0 | 0 |
| 18 | 8824872 | 8824872 | A | T | exonic | SOGA2 | nonsynonymous SNV | 1 | 0 | 0 |
| 12 | 31241977 | 31241977 | G | C | splicing | DDX11 | NAN | 1 | 0 | 0 |
| 17 | 73497198 | 73497198 | T | G | exonic | CASKIN2 | nonsynonymous SNV | 1 | 0 | 0 |
| 4 | 71509845 | 71509845 | G | T | exonic | ENAM | nonsynonymous SNV | 1 | 0 | 0 |
| 17 | 3848412 | 3848412 | A | C | exonic | ATP2A3 | nonsynonymous SNV | 1 | 0 | 0 |
| 7 | 143826349 | 143826349 | C | G | exonic | OR2A14 | nonsynonymous SNV | 1 | 0 | 0 |
| 11 | 111782409 | 111782409 | A | T | exonic | CRYAB | nonsynonymous SNV | 1 | 0 | 0 |
| 1 | 240256578 | 240256578 | A | C | exonic | FMN2 | nonsynonymous SNV | 1 | 0 | 0 |
| 16 | 84456276 | 84456276 | A | T | exonic | ATP2C2 | nonsynonymous SNV | 1 | 0 | 0 |
| 11 | 71729526 | 71729526 | T | G | exonic | NUMA1 | nonsynonymous SNV | 1 | 0 | 0 |
| 20 | 48098869 | 48098869 | T | G | exonic | KCNB1 | nonsynonymous SNV | 1 | 0 | 0 |
| 1 | 41284275 | 41284275 | A | C | exonic | KCNQ4 | nonsynonymous SNV | 1 | 0 | 0 |
| X | 153220965 | 153220965 | A | T | exonic | HCFC1 | nonsynonymous SNV | 1 | 0 | 0 |
| 16 | 56901115 | 56901115 | T | G | exonic | SLC12A3 | nonsynonymous SNV | 1 | 0 | 0 |
| 12 | 76424880 | 76424880 | T | G | exonic | PHLDA1 | nonsynonymous SNV | 1 | 0 | 0 |
| 21 | 47674736 | 47674736 | T | G | exonic | MCM3AP | nonsynonymous SNV | 1 | 0 | 0 |
| 13 | 111372044 | 111372044 | A | C | exonic | ING1 | nonsynonymous SNV | 1 | 0 | 0 |
| X | 48929667 | 48929667 | A | T | exonic | PRAF2 | nonsynonymous SNV | 1 | 0 | 0 |
| 19 | 55598744 | 55598744 | A | C | exonic | EPS8L1 | nonsynonymous SNV | 1 | 0 | 0 |
| 12 | 7556344 | 7556344 | T | C | exonic | CD163L1 | nonsynonymous SNV | 1 | 0 | 0 |
| 15 | 62360922 | 62360922 | A | C | exonic | C2CD4A | stoploss SNV | 1 | 0 | 0 |
| 12 | 40631851 | 40631851 | A | T | exonic | LRRK2 | nonsynonymous SNV | 0 | 1 | 0 |
| 11 | 66358722 | 66358722 | T | C | exonic | CCDC87 | nonsynonymous SNV | 0 | 1 | 0 |
| 3 | 164906182 | 164906182 | C | G | exonic | SLITRK3 | nonsynonymous SNV | 0 | 1 | 0 |
| 19 | 9077598 | 9077598 | A | C | exonic | MUC16 | nonsynonymous SNV | 0 | 1 | 0 |
| 2 | 109545756 | 109545756 | T | A | exonic | EDAR | nonsynonymous SNV | 0 | 1 | 0 |
| 7 | 117188693 | 117188693 | A | T | splicing | CFTR | NAN | 0 | 1 | 0 |
| 12 | 111758299 | 111758299 | A | C | exonic | CUX2 | nonsynonymous SNV | 0 | 1 | 0 |
| 3 | 51978123 | 51978123 | T | A | exonic | PARP3 | nonsynonymous SNV | 0 | 1 | 0 |
| 16 | 71697841 | 71697841 | A | T | exonic | PHLPP2 | stopgain SNV | 0 | 1 | 0 |
| 14 | 21502131 | 21502131 | G | C | exonic | RNASE13 | nonsynonymous SNV | 0 | 1 | 0 |
| 8 | 17823840 | 17823840 | C | G | exonic | PCM1 | nonsynonymous SNV | 0 | 1 | 0 |
| 14 | 44974364 | 44974364 | T | G | exonic | FSCB | nonsynonymous SNV | 0 | 1 | 0 |
| 16 | 74920238 | 74920238 | G | C | exonic | WDR59 | nonsynonymous SNV | 0 | 1 | 0 |
| 12 | 57994660 | 57994660 | T | G | exonic | PIP4K2C | nonsynonymous SNV | 0 | 1 | 0 |
| 9 | 139739734 | 139739734 | T | A | exonic | C9orf172 | nonsynonymous SNV | 0 | 1 | 0 |
| 3 | 69244471 | 69244471 | T | C | exonic | FRMD4B | nonsynonymous SNV | 0 | 1 | 0 |
| 4 | 111469387 | 111469387 | G | C | exonic | ENPEP | nonsynonymous SNV | 0 | 1 | 0 |
| 20 | 60990267 | 60990267 | T | G | exonic | RBBP8NL | nonsynonymous SNV | 0 | 1 | 0 |
| 6 | 152522996 | 152522996 | T | C | exonic | SYNE1 | nonsynonymous SNV | 0 | 1 | 0 |
| 8 | 3200836 | 3200836 | C | G | exonic | CSMD1 | nonsynonymous SNV | 0 | 1 | 0 |
| 1 | 178442271 | 178442271 | T | A | exonic | RASAL2 | nonsynonymous SNV | 0 | 1 | 0 |
| 8 | 106813964 | 106813964 | G | T | exonic | ZFPM2 | stopgain SNV | 0 | 1 | 0 |
| 2 | 231110646 | 231110646 | G | C | exonic | SP140 | nonsynonymous SNV | 0 | 1 | 0 |
| 17 | 41895441 | 41895441 | T | G | exonic | MPP3 | nonsynonymous SNV | 0 | 1 | 0 |
| 17 | 4936268 | 4936268 | T | G | exonic | SLC52A1 | nonsynonymous SNV | 0 | 1 | 0 |
| 2 | 102959548 | 102959548 | C | G | exonic | IL1RL1 | nonsynonymous SNV | 0 | 1 | 0 |
| 19 | 36278522 | 36278522 | T | G | exonic | ARHGAP33 | nonsynonymous SNV | 0 | 1 | 0 |
| 9 | 77563028 | 77563028 | T | G | exonic | C9orf40 | nonsynonymous SNV | 0 | 1 | 0 |
| 22 | 41969697 | 41969697 | T | G | exonic | CSDC2 | nonsynonymous SNV | 0 | 1 | 0 |
| 17 | 40021367 | 40021367 | T | G | exonic | KLHL11 | nonsynonymous SNV | 0 | 1 | 0 |
| 1 | 227152957 | 227152957 | T | G | exonic | ADCK3 | nonsynonymous SNV | 0 | 1 | 0 |
| 15 | 50784950 | 50784950 | C | T | exonic | USP8 | nonsynonymous SNV | 0 | 1 | 0 |
| 22 | 31337957 | 31337957 | T | A | exonic | MORC2 | nonsynonymous SNV | 0 | 1 | 0 |
| 6 | 143094912 | 143094912 | G | C | exonic | HIVEP2 | nonsynonymous SNV | 0 | 1 | 0 |
| 15 | 50784955 | 50784955 | C | A | exonic | USP8 | nonsynonymous SNV | 0 | 1 | 0 |
| 5 | 176952068 | 176952068 | T | G | exonic | FAM193B | nonsynonymous SNV | 0 | 1 | 0 |
| 12 | 57918749 | 57918749 | A | C | exonic | MBD6 | nonsynonymous SNV | 0 | 1 | 0 |
| 2 | 36744469 | 36744469 | G | C | splicing | CRIM1 | NAN | 0 | 1 | 0 |
| 14 | 74416833 | 74416833 | T | G | exonic | COQ6,FAM161B | nonsynonymous SNV | 0 | 1 | 0 |
| 1 | 92646013 | 92646013 | C | T | exonic | KIAA1107 | stopgain SNV | 0 | 1 | 0 |
| 5 | 178358923 | 178358923 | G | T | exonic | ZFP2 | nonsynonymous SNV | 0 | 1 | 0 |
| 6 | 143094935 | 143094935 | G | A | exonic | HIVEP2 | nonsynonymous SNV | 0 | 1 | 0 |
| 17 | 1378247 | 1378247 | T | G | exonic | MYO1C | nonsynonymous SNV | 0 | 1 | 0 |
| X | 47307549 | 47307549 | T | A | exonic | ZNF41 | nonsynonymous SNV | 0 | 1 | 0 |
| 14 | 23596511 | 23596511 | A | T | exonic | SLC7A8 | nonsynonymous SNV | 0 | 1 | 0 |
| 1 | 158913685 | 158913685 | G | C | exonic | PYHIN1 | nonsynonymous SNV | 0 | 1 | 0 |
| 6 | 146993483 | 146993483 | G | C | exonic | ADGB | nonsynonymous SNV | 0 | 1 | 0 |
| 20 | 48599631 | 48599631 | A | C | exonic | SNAI1 | nonsynonymous SNV | 0 | 1 | 0 |
| 11 | 73074259 | 73074259 | A | C | exonic | ARHGEF17 | nonsynonymous SNV | 0 | 1 | 0 |
| 17 | 74133753 | 74133753 | T | G | exonic | FOXJ1 | nonsynonymous SNV | 0 | 1 | 0 |
| 4 | 74735490 | 74735490 | T | A | exonic | CXCL1 | nonsynonymous SNV | 0 | 1 | 0 |
| 8 | 22422003 | 22422003 | A | C | exonic | SORBS3 | nonsynonymous SNV | 0 | 1 | 0 |
| 2 | 196545538 | 196545538 | G | T | exonic | SLC39A10 | nonsynonymous SNV | 0 | 1 | 0 |
| 2 | 98349369 | 98349369 | G | A | exonic | ZAP70 | nonsynonymous SNV | 0 | 1 | 0 |
| 6 | 29641450 | 29641450 | T | G | exonic | ZFP57 | nonsynonymous SNV | 0 | 1 | 0 |
| 22 | 21141269 | 21141269 | A | C | exonic | SERPIND1 | nonsynonymous SNV | 0 | 1 | 0 |
| 7 | 134931294 | 134931294 | A | T | exonic | STRA8 | nonsynonymous SNV | 0 | 1 | 0 |
| 19 | 41040080 | 41040080 | A | C | exonic | SPTBN4 | nonsynonymous SNV | 0 | 1 | 0 |
| 20 | 47260950 | 47260950 | A | C | exonic | PREX1 | nonsynonymous SNV | 0 | 1 | 0 |
| 9 | 34989690 | 34989690 | T | G | exonic | DNAJB5 | nonsynonymous SNV | 0 | 1 | 0 |
| 6 | 160557608 | 160557608 | G | C | exonic | SLC22A1 | nonsynonymous SNV | 0 | 1 | 0 |
| 16 | 90075293 | 90075293 | T | G | exonic | DBNDD1 | nonsynonymous SNV | 0 | 1 | 0 |
| 20 | 18296711 | 18296711 | A | T | exonic | ZNF133 | stopgain SNV | 0 | 1 | 0 |
| 9 | 116346541 | 116346541 | A | C | exonic | RGS3 | nonsynonymous SNV | 0 | 1 | 0 |
| 11 | 1257677 | 1257677 | A | G | exonic | MUC5B | nonsynonymous SNV | 0 | 1 | 0 |
| 16 | 57060531 | 57060531 | A | C | exonic | NLRC5 | nonsynonymous SNV | 0 | 1 | 0 |
| 5 | 178392472 | 178392472 | T | A | exonic | ZNF454 | nonsynonymous SNV | 0 | 1 | 0 |
| 15 | 33445323 | 33445323 | A | T | exonic | FMN1 | nonsynonymous SNV | 0 | 1 | 0 |
| 3 | 45077151 | 45077151 | A | C | exonic | CLEC3B | nonsynonymous SNV | 0 | 1 | 0 |
| 21 | 35286765 | 35286765 | T | A | exonic | ATP5O | stopgain SNV | 0 | 1 | 0 |
| 11 | 122848357 | 122848357 | T | G | exonic | BSX | stoploss SNV | 0 | 1 | 0 |
| 10 | 73562805 | 73562805 | A | C | exonic | CDH23 | nonsynonymous SNV | 0 | 1 | 0 |
| 16 | 67576670 | 67576670 | A | T | exonic | FAM65A | nonsynonymous SNV | 0 | 1 | 0 |
| 8 | 67341490 | 67341490 | T | G | exonic | RRS1 | nonsynonymous SNV | 0 | 1 | 0 |
| 1 | 43893343 | 43893343 | A | C | exonic | SZT2 | nonsynonymous SNV | 0 | 1 | 0 |
| 17 | 41957190 | 41957190 | A | C | exonic | MPP2 | nonsynonymous SNV | 0 | 1 | 0 |
| 7 | 92462408 | 92462408 | A | C | exonic | CDK6 | nonsynonymous SNV | 0 | 1 | 0 |
| X | 77529123 | 77529123 | T | A | exonic | CYSLTR1 | nonsynonymous SNV | 0 | 1 | 0 |
| 19 | 4548317 | 4548317 | A | C | exonic | SEMA6B | nonsynonymous SNV | 0 | 1 | 0 |
| 3 | 49166678 | 49166678 | T | A | exonic | LAMB2 | nonsynonymous SNV | 0 | 1 | 0 |
| X | 22291726 | 22291726 | G | T | exonic | ZNF645 | nonsynonymous SNV | 0 | 1 | 0 |
| 22 | 38041452 | 38041452 | C | T | exonic | SH3BP1 | nonsynonymous SNV | 0 | 1 | 0 |
| 7 | 6661215 | 6661215 | T | A | exonic | ZNF853 | nonsynonymous SNV | 0 | 1 | 0 |
| 5 | 146257710 | 146257710 | A | T | exonic | PPP2R2B | stopgain SNV | 0 | 1 | 0 |
| 5 | 176759235 | 176759235 | T | G | exonic | LMAN2 | nonsynonymous SNV | 0 | 1 | 0 |
| 9 | 117848555 | 117848555 | A | T | exonic | TNC | stopgain SNV | 0 | 1 | 0 |
| 6 | 37442424 | 37442424 | T | G | splicing | CMTR1 | NAN | 0 | 1 | 0 |
| 11 | 17450152 | 17450152 | T | G | exonic | ABCC8 | nonsynonymous SNV | 0 | 1 | 0 |
| 17 | 80274208 | 80274208 | A | T | exonic | CD7 | nonsynonymous SNV | 0 | 1 | 0 |
| 17 | 38178992 | 38178992 | T | G | exonic | MED24 | nonsynonymous SNV | 0 | 1 | 0 |
| 10 | 100195526 | 100195526 | G | A | exonic | HPS1 | nonsynonymous SNV | 0 | 1 | 0 |
| 3 | 49692406 | 49692406 | A | T | exonic | BSN | nonsynonymous SNV | 0 | 1 | 0 |
| 1 | 17296812 | 17296812 | A | C | exonic | CROCC | nonsynonymous SNV | 0 | 1 | 0 |
| 17 | 1375245 | 1375245 | T | G | exonic | MYO1C | nonsynonymous SNV | 0 | 1 | 0 |
| 6 | 1390837 | 1390837 | T | G | exonic | FOXF2 | nonsynonymous SNV | 0 | 1 | 0 |
| X | 153590107 | 153590107 | T | G | exonic | FLNA | nonsynonymous SNV | 0 | 1 | 0 |
| 6 | 36269961 | 36269961 | A | C | exonic | PNPLA1 | nonsynonymous SNV | 0 | 1 | 0 |
| 17 | 76566453 | 76566453 | A | T | exonic | DNAH17 | nonsynonymous SNV | 0 | 1 | 0 |
| 16 | 2027625 | 2027625 | A | C | exonic | TBL3 | nonsynonymous SNV | 0 | 1 | 0 |
| 19 | 13216407 | 13216407 | A | T | exonic | TRMT1 | nonsynonymous SNV | 0 | 1 | 0 |
| 4 | 86491719 | 86491719 | G | A | exonic | ARHGAP24 | nonsynonymous SNV | 0 | 1 | 0 |
| X | 47030490 | 47030490 | A | C | exonic | RBM10 | nonsynonymous SNV | 0 | 1 | 0 |
| 1 | 205538415 | 205538415 | T | G | exonic | MFSD4 | nonsynonymous SNV | 0 | 1 | 0 |
| 6 | 35255432 | 35255432 | A | C | exonic | ZNF76 | nonsynonymous SNV | 0 | 1 | 0 |
| 8 | 143747260 | 143747260 | T | G | exonic | JRK | nonsynonymous SNV | 0 | 1 | 0 |
| 1 | 227169797 | 227169797 | T | G | exonic | ADCK3 | nonsynonymous SNV | 0 | 1 | 0 |
| 1 | 181702833 | 181702833 | T | G | exonic | CACNA1E | nonsynonymous SNV | 0 | 1 | 0 |
| 11 | 17449954 | 17449954 | T | A | splicing | ABCC8 | NAN | 0 | 1 | 0 |
| 2 | 232262681 | 232262681 | T | A | exonic | B3GNT7 | nonsynonymous SNV | 0 | 1 | 0 |
| 14 | 34269590 | 34269590 | T | C | exonic | NPAS3 | nonsynonymous SNV | 0 | 1 | 0 |
| 6 | 108492725 | 108492725 | T | G | exonic | NR2E1 | nonsynonymous SNV | 0 | 1 | 0 |
| 19 | 15224650 | 15224650 | T | G | exonic | SYDE1 | nonsynonymous SNV | 0 | 1 | 0 |
| 20 | 60905945 | 60905945 | C | T | exonic | LAMA5 | nonsynonymous SNV | 0 | 1 | 0 |
| 1 | 201252852 | 201252852 | A | C | exonic | PKP1 | nonsynonymous SNV | 0 | 1 | 0 |
| 10 | 111667573 | 111667573 | T | G | exonic | XPNPEP1 | nonsynonymous SNV | 0 | 1 | 0 |
| 10 | 94824220 | 94824220 | C | T | exonic | CYP26C1 | nonsynonymous SNV | 0 | 1 | 0 |
| 11 | 66360389 | 66360389 | T | G | exonic | CCDC87 | nonsynonymous SNV | 0 | 1 | 0 |
| 17 | 42433582 | 42433582 | A | T | exonic | FAM171A2 | nonsynonymous SNV | 0 | 1 | 0 |
| 2 | 29293682 | 29293682 | G | A | exonic | C2orf71 | nonsynonymous SNV | 0 | 1 | 0 |
| 22 | 20920853 | 20920853 | T | A | exonic | MED15 | nonsynonymous SNV | 0 | 1 | 0 |
| 5 | 169504763 | 169504763 | C | G | exonic | DOCK2 | stopgain SNV | 0 | 1 | 0 |
| 12 | 49426692 | 49426692 | T | A | exonic | KMT2D | nonsynonymous SNV | 0 | 1 | 0 |
| 17 | 3629177 | 3629177 | T | A | exonic | GSG2 | nonsynonymous SNV | 0 | 1 | 0 |
| 4 | 3444589 | 3444589 | T | G | exonic | HGFAC | nonsynonymous SNV | 0 | 1 | 0 |
| 10 | 73767228 | 73767228 | T | G | exonic | CHST3 | nonsynonymous SNV | 0 | 1 | 0 |
| 6 | 88757627 | 88757627 | A | C | exonic | SPACA1 | nonsynonymous SNV | 0 | 1 | 0 |
| 1 | 71513251 | 71513251 | T | G | exonic | PTGER3 | nonsynonymous SNV | 0 | 1 | 0 |
| 19 | 13919659 | 13919659 | A | C | exonic | ZSWIM4 | nonsynonymous SNV | 0 | 1 | 0 |
| 12 | 133249259 | 133249259 | A | C | exonic | POLE | nonsynonymous SNV | 0 | 1 | 0 |
| 15 | 69320688 | 69320688 | A | T | exonic | NOX5 | nonsynonymous SNV | 0 | 1 | 0 |
| 19 | 3548195 | 3548195 | A | C | exonic | MFSD12 | nonsynonymous SNV | 0 | 1 | 0 |
| 12 | 53880986 | 53880986 | T | G | exonic | MAP3K12 | nonsynonymous SNV | 0 | 1 | 0 |
| 10 | 23729189 | 23729189 | A | C | exonic | OTUD1 | nonsynonymous SNV | 0 | 1 | 0 |
| 19 | 54932515 | 54932515 | T | G | exonic | TTYH1 | nonsynonymous SNV | 0 | 1 | 0 |
| 21 | 46067148 | 46067148 | C | A | exonic | KRTAP10-11 | nonsynonymous SNV | 0 | 1 | 0 |
| 19 | 39993499 | 39993499 | T | G | exonic | DLL3 | nonsynonymous SNV | 0 | 1 | 0 |
| 9 | 71629007 | 71629007 | A | T | exonic | PRKACG | nonsynonymous SNV | 0 | 1 | 0 |
| 12 | 80083693 | 80083693 | T | G | exonic | PAWR | nonsynonymous SNV | 0 | 1 | 0 |
| 19 | 36124791 | 36124791 | A | C | exonic | RBM42 | nonsynonymous SNV | 0 | 1 | 0 |
| 12 | 53298699 | 53298699 | G | A | exonic | KRT8 | nonsynonymous SNV | 0 | 1 | 0 |
| 2 | 186696574 | 186696574 | T | G | exonic | FSIP2 | nonsynonymous SNV | 0 | 1 | 0 |
| 12 | 100169415 | 100169415 | G | T | exonic | ANKS1B | nonsynonymous SNV | 0 | 1 | 0 |
| 8 | 145578369 | 145578369 | A | C | exonic | TMEM249 | nonsynonymous SNV | 0 | 0 | 1 |
| 7 | 4830973 | 4830973 | T | G | exonic | AP5Z1 | nonsynonymous SNV | 0 | 0 | 1 |
| 7 | 1517235 | 1517235 | A | C | exonic | INTS1 | nonsynonymous SNV | 0 | 0 | 1 |
| 16 | 71686865 | 71686865 | G | T | exonic | PHLPP2 | nonsynonymous SNV | 0 | 0 | 1 |
| 10 | 124697633 | 124697633 | T | A | exonic | C10orf88 | nonsynonymous SNV | 0 | 0 | 1 |
| 16 | 67063708 | 67063708 | T | G | exonic | CBFB | nonsynonymous SNV | 0 | 0 | 1 |
| 1 | 156646380 | 156646380 | T | G | exonic | NES | nonsynonymous SNV | 0 | 0 | 1 |
| 11 | 67815038 | 67815038 | A | C | exonic | TCIRG1 | nonsynonymous SNV | 0 | 0 | 1 |
| 19 | 42853690 | 42853690 | A | C | exonic | MEGF8 | nonsynonymous SNV | 0 | 0 | 1 |
| 7 | 150769027 | 150769027 | A | T | splicing | SLC4A2 | NAN | 0 | 0 | 1 |
| 4 | 122872685 | 122872685 | A | C | exonic | TRPC3 | nonsynonymous SNV | 0 | 0 | 1 |
| 1 | 16360128 | 16360128 | T | A | exonic | CLCNKA | nonsynonymous SNV | 0 | 0 | 1 |
| 14 | 105617223 | 105617223 | A | C | exonic | JAG2 | nonsynonymous SNV | 0 | 0 | 1 |
| 20 | 23432550 | 23432550 | T | G | exonic | CST11 | nonsynonymous SNV | 0 | 0 | 1 |
| 4 | 1165847 | 1165847 | T | G | exonic | SPON2 | nonsynonymous SNV | 0 | 0 | 1 |
| 12 | 132516687 | 132516687 | A | T | exonic | EP400 | nonsynonymous SNV | 0 | 0 | 1 |
| 8 | 52321884 | 52321884 | A | C | exonic | PXDNL | nonsynonymous SNV | 0 | 0 | 1 |
| 1 | 152883220 | 152883220 | T | A | exonic | IVL | nonsynonymous SNV | 0 | 0 | 1 |
| 5 | 61875705 | 61875705 | T | C | exonic | LRRC70 | nonsynonymous SNV | 0 | 0 | 1 |
| 1 | 11884584 | 11884584 | T | G | exonic | CLCN6 | nonsynonymous SNV | 0 | 0 | 1 |
| 19 | 38905700 | 38905700 | A | T | exonic | RASGRP4 | nonsynonymous SNV | 0 | 0 | 1 |
| 9 | 101765733 | 101765733 | A | T | splicing | COL15A1 | NAN | 0 | 0 | 1 |
| 20 | 17417386 | 17417386 | A | C | exonic | PCSK2 | nonsynonymous SNV | 0 | 0 | 1 |
| 4 | 3230689 | 3230689 | T | G | exonic | HTT | nonsynonymous SNV | 0 | 0 | 1 |
| 1 | 17596751 | 17596751 | T | A | exonic | PADI3 | nonsynonymous SNV | 0 | 0 | 1 |
| 7 | 128496939 | 128496939 | T | G | exonic | FLNC | nonsynonymous SNV | 0 | 0 | 1 |
| 1 | 21044063 | 21044063 | T | G | exonic | KIF17 | nonsynonymous SNV | 0 | 0 | 1 |
| 16 | 69402344 | 69402344 | A | T | exonic | TERF2 | nonsynonymous SNV | 0 | 0 | 1 |
| 17 | 3599274 | 3599274 | A | C | exonic | P2RX5 | nonsynonymous SNV | 0 | 0 | 1 |
| 17 | 76130991 | 76130991 | T | G | exonic | TMC8 | nonsynonymous SNV | 0 | 0 | 1 |
| 8 | 22548972 | 22548972 | T | G | exonic | EGR3 | nonsynonymous SNV | 0 | 0 | 1 |
| 7 | 100084521 | 100084521 | T | G | exonic | NYAP1 | nonsynonymous SNV | 0 | 0 | 1 |
| 17 | 8141789 | 8141789 | T | G | exonic | CTC1 | nonsynonymous SNV | 0 | 0 | 1 |
| 19 | 35524942 | 35524942 | A | T | exonic | SCN1B | nonsynonymous SNV | 0 | 0 | 1 |
| 11 | 47609721 | 47609721 | G | A | exonic | FAM180B | nonsynonymous SNV | 0 | 0 | 1 |
| 14 | 24884291 | 24884291 | A | C | exonic | NYNRIN | nonsynonymous SNV | 0 | 0 | 1 |
| 1 | 156616700 | 156616700 | A | C | exonic | BCAN | nonsynonymous SNV | 0 | 0 | 1 |
| 14 | 23346447 | 23346447 | T | G | exonic | LRP10 | nonsynonymous SNV | 0 | 0 | 1 |
| 3 | 97668717 | 97668717 | G | A | exonic | MINA | nonsynonymous SNV | 0 | 0 | 1 |
| 6 | 32083584 | 32083584 | A | T | exonic | ATF6B | nonsynonymous SNV | 0 | 0 | 1 |
| X | 114426485 | 114426485 | T | A | exonic | RBMXL3 | nonsynonymous SNV | 0 | 0 | 1 |
| 20 | 43052739 | 43052739 | A | C | exonic | HNF4A | nonsynonymous SNV | 0 | 0 | 1 |
| 6 | 43144351 | 43144351 | T | G | exonic | SRF | nonsynonymous SNV | 0 | 0 | 1 |
| 1 | 107599660 | 107599660 | T | G | exonic | PRMT6 | nonsynonymous SNV | 0 | 0 | 1 |
| 10 | 49658430 | 49658430 | T | G | exonic | ARHGAP22 | nonsynonymous SNV | 0 | 0 | 1 |
| 1 | 6648211 | 6648211 | A | C | exonic | ZBTB48 | nonsynonymous SNV | 0 | 0 | 1 |
| 22 | 31495104 | 31495104 | A | C | exonic | SMTN | nonsynonymous SNV | 0 | 0 | 1 |
| 3 | 37067492 | 37067492 | A | T | exonic | MLH1 | nonsynonymous SNV | 0 | 0 | 1 |
| 9 | 37745852 | 37745852 | T | A | exonic | FRMPD1 | nonsynonymous SNV | 0 | 0 | 1 |
| 2 | 233394805 | 233394805 | T | G | exonic | CHRND | nonsynonymous SNV | 0 | 0 | 1 |
| 1 | 204214063 | 204214063 | T | G | exonic | PLEKHA6 | nonsynonymous SNV | 0 | 0 | 1 |
| 3 | 39144291 | 39144291 | T | G | exonic | GORASP1 | nonsynonymous SNV | 0 | 0 | 1 |
| 11 | 57427409 | 57427409 | A | T | exonic | CLP1 | nonsynonymous SNV | 0 | 0 | 1 |
| 1 | 145532248 | 145532248 | A | C | exonic | ITGA10 | nonsynonymous SNV | 0 | 0 | 1 |
| 12 | 110778561 | 110778561 | T | G | exonic | ATP2A2 | nonsynonymous SNV | 0 | 0 | 1 |
| 1 | 27939613 | 27939613 | A | T | exonic | FGR | nonsynonymous SNV | 0 | 0 | 1 |
| 18 | 3457612 | 3457612 | T | G | exonic | TGIF1 | nonsynonymous SNV | 0 | 0 | 1 |
| 16 | 1841112 | 1841112 | A | C | exonic | IGFALS | nonsynonymous SNV | 0 | 0 | 1 |
| 3 | 184100942 | 184100942 | A | C | exonic | CHRD | nonsynonymous SNV | 0 | 0 | 1 |
| 19 | 13919733 | 13919733 | A | C | exonic | ZSWIM4 | nonsynonymous SNV | 0 | 0 | 1 |
| 7 | 149485054 | 149485054 | T | G | exonic | SSPO | nonsynonymous SNV | 0 | 0 | 1 |
| 19 | 38886247 | 38886247 | T | G | exonic | SPRED3 | nonsynonymous SNV | 0 | 0 | 1 |
| 12 | 49317603 | 49317603 | A | T | exonic | FKBP11 | stopgain SNV | 0 | 0 | 1 |
| 7 | 4116823 | 4116823 | T | G | splicing | SDK1 | NAN | 0 | 0 | 1 |
| 21 | 47987402 | 47987402 | T | G | exonic | DIP2A | nonsynonymous SNV | 0 | 0 | 1 |
| 19 | 17887488 | 17887488 | A | T | exonic | FCHO1 | nonsynonymous SNV | 0 | 0 | 1 |
| 2 | 133489565 | 133489565 | A | C | exonic | NCKAP5 | nonsynonymous SNV | 0 | 0 | 1 |
| 10 | 102769011 | 102769011 | A | C | exonic | PDZD7 | nonsynonymous SNV | 0 | 0 | 1 |
| 17 | 79096403 | 79096403 | A | C | exonic | AATK | nonsynonymous SNV | 0 | 0 | 1 |
| 11 | 1263094 | 1263094 | T | A | exonic | MUC5B | nonsynonymous SNV | 0 | 0 | 1 |
| 17 | 18025090 | 18025090 | A | C | exonic | MYO15A | nonsynonymous SNV | 0 | 0 | 1 |
| 2 | 175618461 | 175618461 | T | G | exonic | CHRNA1 | nonsynonymous SNV | 0 | 0 | 1 |
| 16 | 50332939 | 50332939 | T | G | exonic | ADCY7 | nonsynonymous SNV | 0 | 0 | 1 |
| 7 | 99705733 | 99705733 | A | T | exonic | TAF6 | nonsynonymous SNV | 0 | 0 | 1 |
| 1 | 6214930 | 6214930 | T | G | exonic | CHD5 | nonsynonymous SNV | 0 | 0 | 1 |
| 12 | 54109587 | 54109587 | T | A | exonic | CALCOCO1 | nonsynonymous SNV | 0 | 0 | 1 |
| 16 | 72821754 | 72821754 | A | C | exonic | ZFHX3 | nonsynonymous SNV | 0 | 0 | 1 |
| 1 | 2238179 | 2238179 | A | C | exonic | SKI | nonsynonymous SNV | 0 | 0 | 1 |
| 15 | 101440785 | 101440785 | T | A | exonic | ALDH1A3 | nonsynonymous SNV | 0 | 0 | 1 |
| 17 | 72308281 | 72308281 | T | G | exonic | DNAI2 | nonsynonymous SNV | 0 | 0 | 1 |
| 6 | 43473219 | 43473219 | T | G | exonic | TJAP1 | nonsynonymous SNV | 0 | 0 | 1 |
| 1 | 150789283 | 150789283 | G | A | exonic | ARNT | nonsynonymous SNV | 0 | 0 | 1 |
| 14 | 39901278 | 39901278 | A | C | exonic | FBXO33 | nonsynonymous SNV | 0 | 0 | 1 |
| 16 | 57075906 | 57075906 | T | A | exonic | NLRC5 | nonsynonymous SNV | 0 | 0 | 1 |
| 10 | 47701157 | 47701157 | A | T | exonic | ANTXRL | nonsynonymous SNV | 0 | 0 | 1 |
| 17 | 77809161 | 77809161 | T | G | exonic | CBX4 | nonsynonymous SNV | 0 | 0 | 1 |
| 18 | 60191388 | 60191388 | A | C | exonic | ZCCHC2 | nonsynonymous SNV | 0 | 0 | 1 |
| 1 | 165797126 | 165797126 | A | C | exonic | UCK2 | nonsynonymous SNV | 0 | 0 | 1 |
| 1 | 152082274 | 152082274 | T | A | exonic | TCHH | nonsynonymous SNV | 0 | 0 | 1 |
| 5 | 133901840 | 133901840 | T | A | exonic | JADE2 | nonsynonymous SNV | 0 | 0 | 1 |
| 1 | 157738462 | 157738462 | T | G | exonic | FCRL2 | nonsynonymous SNV | 0 | 0 | 1 |
| 11 | 1102496 | 1102496 | A | T | exonic | MUC2 | nonsynonymous SNV | 0 | 0 | 1 |
| 16 | 70285206 | 70285206 | T | G | exonic | EXOSC6 | nonsynonymous SNV | 0 | 0 | 1 |
| 14 | 56763334 | 56763334 | A | C | exonic | PELI2 | nonsynonymous SNV | 0 | 0 | 1 |
| 19 | 7676818 | 7676818 | A | C | exonic | CAMSAP3 | nonsynonymous SNV | 0 | 0 | 1 |
| 20 | 61938840 | 61938840 | A | C | splicing | COL20A1 | NAN | 0 | 0 | 1 |
| 17 | 73913865 | 73913865 | A | C | exonic | FBF1 | nonsynonymous SNV | 0 | 0 | 1 |
| 3 | 126733067 | 126733067 | T | G | exonic | PLXNA1 | nonsynonymous SNV | 0 | 0 | 1 |
| 11 | 64603610 | 64603610 | T | G | exonic | CDC42BPG | nonsynonymous SNV | 0 | 0 | 1 |
| 1 | 155172179 | 155172179 | T | G | exonic | THBS3 | nonsynonymous SNV | 0 | 0 | 1 |
| 20 | 23066784 | 23066784 | T | G | exonic | CD93 | nonsynonymous SNV | 0 | 0 | 1 |
| 4 | 48492459 | 48492459 | T | G | exonic | ZAR1 | nonsynonymous SNV | 0 | 0 | 1 |
| 17 | 36508579 | 36508579 | T | A | exonic | SOCS7 | nonsynonymous SNV | 0 | 0 | 1 |
| 19 | 50376279 | 50376279 | T | G | exonic | AKT1S1 | nonsynonymous SNV | 0 | 0 | 1 |
| 3 | 126219587 | 126219587 | T | G | exonic | UROC1 | nonsynonymous SNV | 0 | 0 | 1 |
| 12 | 53818561 | 53818561 | A | C | exonic | AMHR2 | nonsynonymous SNV | 0 | 0 | 1 |
| 14 | 38678643 | 38678643 | A | C | exonic | SSTR1 | nonsynonymous SNV | 0 | 0 | 1 |
| 2 | 128051114 | 128051114 | T | G | exonic | ERCC3 | nonsynonymous SNV | 0 | 0 | 1 |
| 17 | 3716411 | 3716411 | C | T | exonic | C17orf85 | nonsynonymous SNV | 0 | 0 | 1 |
| 10 | 81925854 | 81925854 | T | A | exonic | ANXA11 | stopgain SNV | 0 | 0 | 1 |
| 5 | 66460796 | 66460796 | A | C | exonic | MAST4 | nonsynonymous SNV | 0 | 0 | 1 |
| 7 | 100137133 | 100137133 | T | G | exonic | AGFG2 | nonsynonymous SNV | 0 | 0 | 1 |
| 19 | 14167242 | 14167242 | T | A | exonic | PALM3 | nonsynonymous SNV | 0 | 0 | 1 |
| 3 | 134089701 | 134089701 | T | G | exonic | AMOTL2 | nonsynonymous SNV | 0 | 0 | 1 |
| 3 | 173997429 | 173997429 | T | A | exonic | NLGN1 | nonsynonymous SNV | 0 | 0 | 1 |
| 2 | 163212989 | 163212989 | G | C | exonic | GCA | nonsynonymous SNV | 0 | 0 | 1 |
| 15 | 20740295 | 20740295 | A | C | exonic | GOLGA6L6 | nonsynonymous SNV | 0 | 0 | 1 |

“0” represents undetected SNVs.

“1” represents detected SNVs.
